# DcLcyB1 and DcLcyB2, two lycopene β-cyclases with partial functional overlap modulate carotene profiles in carrot roots via distinct catalytic properties

**DOI:** 10.1186/s43897-026-00232-z

**Published:** 2026-07-01

**Authors:** Ya-Hui Wang, Pei-Zhuo Liu, Rong-Rong Zhang, Yu-Qing Zhang, Hui-Ru Wang, Yu-Jie Sun, Jing Ma, Zhi-Sheng Xu, Ai-Sheng Xiong

**Affiliations:** 1 State Key Laboratory of Crop Genetics & Germplasm Enhancement and Utilization, Ministry of Agriculture and Rural Affairs Key Laboratory of Biology and Germplasm Enhancement of Horticultural Crops in East China, College of Horticulture, Nanjing Agricultural University, 1 Weigang, Nanjing, Jiangsu 210095 China; 2 College of Horticulture, Qingdao Agricultural University, Qingdao, Shandong 266109 China

**Keywords:** Carotenes, Coexpression, Cyclization, Lycopene β-cyclase, Carrot

## Abstract

In plant, lycopene β-cyclase is a crucial enzyme in carotenoid metabolic pathway, which can induce color alteration. Carrots (*Daucus carota*) possess two genes encoding lycopene β-cyclase, namely *DcLcyB1* and *DcLcyB2*. Little is known regarding the functional disparities between these two proteins in regulating carrot carotenoid accumulation. We found that the expression level of *DcLcyB2* was higher than that of *DcLcyB1* in carrot roots. Enzyme reaction in *E. coli* demonstrated that both two DcLcyB proteins were capable of cycloconverting lycopene to β-carotene, but DcLcyB2 tended to have a higher preference for monocyclic carotene substrates, resulting in more α-carotene production. After the *DcLcyBs* were separately overexpressed in red carrots, the roots turned yellow, accompanied by the reduction of lycopene and β-carotene content and the entire carotenoid metabolism flowed downstream towards xanthophylls. The expression levels of *DcCHXE*, *DcCYP97A3*, *DcCHXB1* and *DcCHXB2* in the *DcLcyB-OE* lines raised sharply. After gene editing of DcLcyB1/2, the α-/β-carotene ratio changed conspicuously, particularly in the *dclcyb2* mutants, where α-carotene content dropped sharply, while β-carotene remained high. The expression levels of most structural genes in carotenoid pathway responded dynamically. Our results enriched the understanding of functionally redundant but differentiated roles of two DcLcyB isoenzymes in carrot root coloring.

**Supplementary Information:**

The online version contains supplementary material available at 10.1186/s43897-026-00232-z.

## Core

In carrot, two DcLcyB isoenzymes exhibited partial functional redundancy while showing differentiation in their cyclization activity. DcLcyB2 held a stronger substrate preference for monocyclic carotene, which affected the ratio of α-carotene and β-carotene. Enhancing *DcLCYB* expression in red carrot promoted the co-expression of carotene hydroxylase gene and led to metabolic flow to downward xanthophylls.

## Gene & accession numbers

**Fig. 1:**
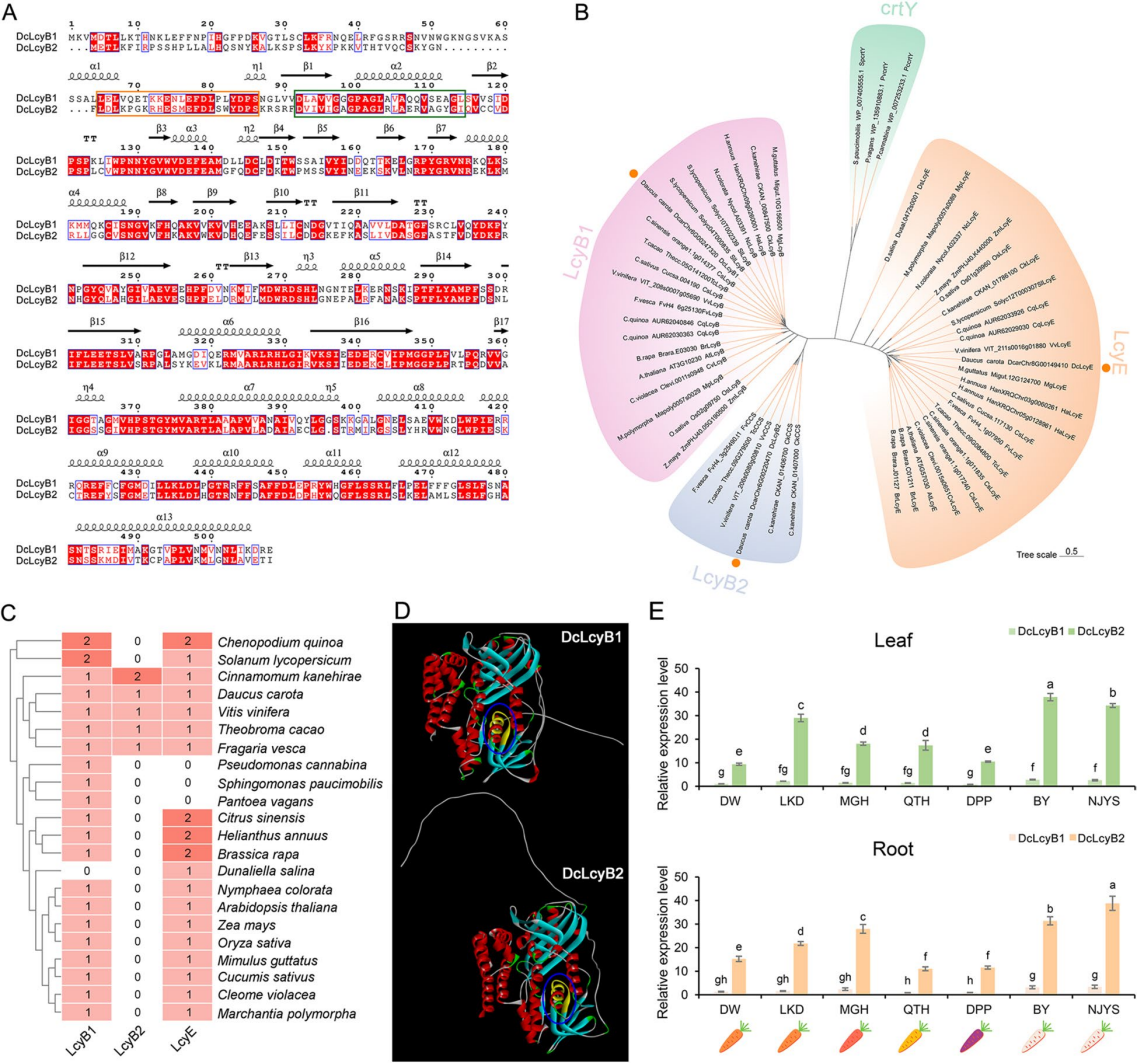
Identification of carrot DcLcyB proteins. **A** Amino acid sequence alignment of DcLcyB1 and DcLcyB2 proteins. The amino acid position is numerically marked. The orange and green boxes represent LcyB protein conserved region and dinucleotide binding site, respectively. The arrows and spiral marks represent the protein secondary structure. **B** Phylogenetic tree of lycopene cyclase proteins in different species. Different proteins are labeled with the species name, gene ID, and name. The carrot lycopene cyclase proteins are pointed out by orange dots. The lower right scale represents genetic distance. *Arabidopsis thaliana*, *A. thaliana*; *Brassica rapa*, *B. rapa*; *Chenopodium quinoa*, *C. quinoa*; *Cinnamomum kanehirae*, *C. kanehirae*; *Citrus sinensis*, *C. sinesis*; *Cleome violacea*, *C. violacea*; *Cucumis sativus*, *C. sativus*; *Dunaliella salina*, *D. salina*; *Fragaria vesca*, *F. vesca*; *Helianthus annuus*, *H. annuus*; *Marchantia polymorpha*, *M. polymorpha*; *Mimulus guttatus*, *M. guttatus*; *Nymphaea colorata*, *N. colorata*; *Oryza sativa*, *O. sativa*; *Pantoea vagans*, *P. vagans*; *Pseudomonas cannabina*, *P. cannabina*; *Solanum lycopersicum*, *S. lycopersicum*; *Sphingomonas paucimobilis*, *S. paucimobilis*; *Theobroma cacao*, *T. cacao*; *Vitis vinifera*, *V. vinifera*; *Zea mays*, *Z. mays*. **C** Number of lycopene cyclase genes in carrot and other 21 species. **D** Prediction of the tertiary structure of DcLcyB proteins. Red, light blue and green represent α-helix, β-sheet and β-turn, respectively. The yellow highlighted amino acid residues with the blue ellipse indicate the conserved motif V/IXGXGXXGXXXA responsible for cofactor binding to the LcyB protein. **E** Relative expression levels of *DcLcyB* genes in leaves and roots of different carrot cultivars. The significance analysis was determined by single factor ANOVA method and multiple comparison was performed by least significant difference (LSD) method. Different lowercase letters represent significant difference at 0.05 level

Sequence of carrot genes in this study were from the Daucus carota T2T genome (NCBI GenBank No.: GCA_030127425.1). The gene ID of DcLcyB1 and DcLcyB2 were DcarChr6G00247320 and DcarChr6G00220470, respectively. The LcyB and LcyE protein sequences of other species were obtained from Phytozome database (https://phytozome-next.jgi.doe.gov/), and the gene ID were marked in the Figure 1.

## Introduction

Carotenoids are prevalently found in all photosynthetic organisms as well as some non-photosynthetic prokaryotes, fungi, and a few animals (Caferri et al 2022; Liu et al 2021; Maoka 2020; Toomey et al 2022). In photosynthetic organisms, carotenoids are essential pigments for photosynthesis and photoprotection, similar to chlorophyll (Muzzopappa & Kirilovsky 2020). Concurrently, they also exert functions in lipid protection and reactive oxygen removal. Carotenoids with epoxide groups are essential for photosynthetic organisms. If the ring structure of carotenoids could not be formed, the organism might encounter death. This also corresponds to why carotenes and xanthophylls are widely discovered in plants (Cunningham et al 1996).

After the action of several catalytic enzymes, the final substance of the straight chain carotenoid, lycopene, is formed. Here also ushered in a crucial branch point of carotenoid biosynthesis pathway, lycopene cyclization (Wang et al 2023a,b). The cyclization of lycopene is accomplished by lycopene cyclase. Different types of lycopene cyclase form a ring structure (ɛ- or β-ring) at the both ends of lycopene to produce cyclic carotenes mainly β-carotene, as α-carotene accumulates mainly in plants and certain algae (Takaichi 2011). Lycopene cyclase is encoded by the *crtY* gene in non-photosynthetic and anaerobic photosynthetic bacteria and is relatively conserved. In organisms with oxygenic photosynthesis, lycopene cyclase is encoded by *crtL* (Sieiro et al 2003). The carotenoid pathway in fungi is relatively simple, where lycopene cyclase and phytoene synthase are encoded by a single gene to produce a bifunctional enzyme similar to *crtY* and *crtB* (Wang et al 2021). Lycopene cyclase in plants can be divided into lycopene ɛ-cyclase (LcyE) and lycopene β-cyclase (LcyB), which are respectively accountable for the formation of the ɛ-ring and β-ring (Koc et al 2015). Along with the evolution and expansion of plant genomes, lycopene cyclase genes have gradually undergone multiple duplication events, and their functions have diverged, being either redundancy or complementarity.

Many efforts have been exerted to investigate the function of lycopene cyclase. Microbial lycopene cyclase is frequently used in microbial cell factories for production of carotenoids, especially β-carotene (Huang et al 2023). To reveal the chemical mechanism, substrate specificity, and activity of lycopene cyclase, a large number of experiments have been conducted and further applied to the optimization of carotenoid synthesis technology (Tokunaga et al 2021; Xie et al 2024; Yu et al 2022). Researches on the function of plant lycopene β-cyclase mainly focus on two aspects: carotenoid accumulation and stress resistance. Studies in tobacco (*Nicotiana tabacum*) (Shi et al 2015), saffron (*Crocus sativus*) (Mir et al 2024), wheat (*Triticum turgidum*) (Yu et al 2022), sweet potato (*Ipomoea batatas*) (Kang et al 2018) and other plants have manifested that *LcyB* overexpression plants or mutants could enhance the content of carotenoids and the ability to withstand stresses such as salt and drought. In watermelon (*Citrullus lanatus*), ClLcyB protein encodes a *rf* site that governs watermelon flesh color, and decreased abundance of LcyB protein leads to the red color of the flesh (Zhang et al 2020). In carrots, Jo et al (2021) discovered that an amino acid insertion of LCYB2 could cause the accumulation of lycopene in carrot root. Experiments in tobacco also proved that both carrot *DcLCYB1* and *DcLCYB2* could enhance stress resistance by regulating carotenoid, gibberellin and chlorophyll pathways, and the plant growth and hormone metabolism of transgenic tobacco were also altered (Kossler et al 2021; Moreno et al 2016, 2013; Rosas-Saavedra et al 2023).

Carrots (*Daucus carota* L.) are among the most typical plants that accumulate abundant carotenoids (Que et al 2019; Wang et al 2022). The common carrots with orange fleshy roots accumulate considerable amounts of α-carotene and β-carotene (Coe et al 2021). A total of two DcLcyB proteins have been identified in carrot (Wang et al 2023a). Although definite progress has been achieved in the lycopene β-cyclase in carrot, the functional differences between the two DcLcyB proteins in carotenoid accumulation remain poorly understood. Recent studies have revealed that the DcLcyE protein regulates the α-branch metabolic flow of the carrot carotenoid pathway (Wang et al 2023c), but whether it is related to DcLcyB awaits further exploration. Therefore, in this study, we isolated two carrot *DcLcyB* genes, *DcLcyB1* and *DcLcyB2*. The catalytic activity of DcLcyBs was analyzed, and overexpression and gene editing were respectively conducted in carrot to disclose its mechanism of action in carotenoid accumulation in carrots, and to clarify the functional differences and significances of the two DcLcyB proteins. The results of this study will complement the understanding of carrot lycopene cyclase, deepen the insights of carrot carotenoid accumulation mechanism, and furnish a foundation for the directional cultivation of carrot germplasm resources rich in vitamin A precursors.

## Results

### Identification of two lycopene β-cyclase genes in carrot

According to the annotated information of the carrot genome, two genes, *DcLcyB1* and *DcLcyB2*, encoding lycopene β-cyclase in carrot, were identified. The two *DcLcyB* genes were cloned from the carrot ‘Kurodagosun’ (KRD), and full-length open reading frame (ORF) of 1,527 and 1,479 bp were obtained, respectively. The amino acid sequence of the two DcLcyB proteins achieved a consistency of 52.17%, and the discrepant sequences were mainly located at the N-terminus of the proteins. Protein structure prediction indicated that the carrot DcLcyB protein comprised 13 α-helixes, 16 β-sheets and 5 β-turns (Fig. 1A). Both two DcLcyB proteins contained the conserved region and dinucleotide binding site of lycopene cyclase protein. We compared the amino acid sequence differences of DcLcyB1 and DcLcyB2 proteins in different carrot cultivars, ‘Deep Purple’ (DPP), ‘Nanjingyesheng’ (NJYS), ‘Baiyu’ (BY), ‘Leikende’ (LKD), ‘Meiguihong’ (MGH), ‘Qitouhuang’ (QTH), and KRD, and the results indicated that the DcLcyB1 protein contained 508 amino acids, and differential sites were found at position 44, 45, 68, 115, 167, 385, and 401 (Figure S1). Similarly, the results of protein sequence comparison among different cultivars of DcLcyB2 displayed that the DcLcyB2 protein contained 492 amino acids, among which there were 5 distinct sites distributed at the positions 7, 25, 92, 338, and 423 (Figure S2).

### Evolution of lycopene cyclase proteins

Based on protein sequence homology, we identified lycopene protein (including LcyE and LcyB) in *D. carota* and 21 other species. These species encompassed photosynthetic bacteria, algae, mosses and monocotyledonous and dicotyledonous plants (Fig. 1B, C). Only one lycopene cyclase, commonly known as crtY, was identified in the bacteria, and the evolutionary tree indicated that they clustered in a single strand. In *D. salina*, only one lycopene cyclase protein was found as well, but it was more closely related to LcyE. The lycopene cyclase in higher plants is functionally differentiated into LcyB and LcyE, and LcyB can be further divided into LcyB1 and LcyB2. In some higher plants that accumulate capsanthin, the specific LcyB enzyme responsible for the synthesis of capsanthin, which also possesses the β-cyclization function, is also names as capsanthin-capsorubin synthase (CCS). Carrots had one each of DcLcyB1, DcLcyB2 and DcLcyE, the same amount as *V. vinifera*, *T. cacao*, *F. vesca*. Among them, DcLcyB1 was more closely related to two SlLcyBs. To ensure whether there was functional bias between the two DcLcyB proteins at the protein structure level, their tertiary structures were determined (Fig. 1D). The results demonstrated that the protein structure of the two proteins were similar, and both contained a cofactor (FAD or NAD(P)) binding fold formed by the conserved motif V/IXGXGXXGXXXA, which was begun with a β-turn connected by an α-helix.

### Differential expression of *DcLcyB* genes in carrot

To explore the potential function of carrot *DcLcyB* genes, we compared the expression levels of two *DcLcyB* genes in carrots with different color roots (Fig. 1E). Both in the leaves and roots, the expression level of *DcLcyB2* was significantly higher than that of *DcLcyB1* in all the carrot cultivars, with a difference multiple of about 10 times. The selected carrot varieties contained 5 root colors of red, orange, yellow, white, and purple, respectively. Among them, the expression levels of *DcLcyB1* and *DcLcyB2* in white root carrot ‘BY’ and ‘NJYS’ were the highest among all the cultivars. On the contrary, the expression levels were the lowest in ‘QTH’ and ‘DPP’. The expression level of *DcLcyB1* in ‘QTH’ roots was only 0.25 times that of ‘NJYS’, and *DcLcyB2* was 0.28 times.

### Analysis of DcLcyB protein catalytic activity

**Fig. 2:**
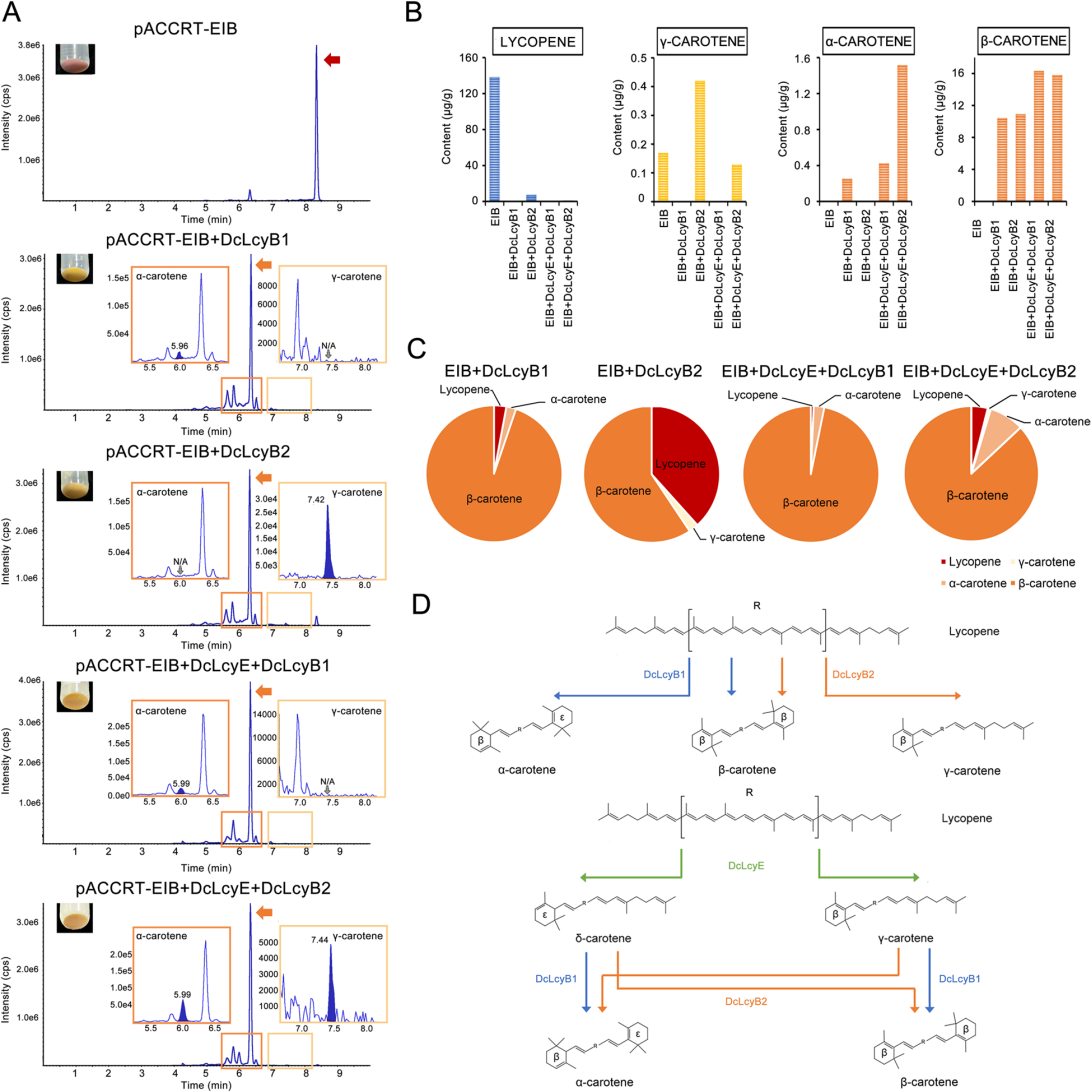
Analysis of catalytic activity of DcLCYB proteins by heterologous expression in *E. coli*. **A** Total ion chromatography of carotenoid extraction from *E. coli*. The upper left corner shows the *E. coli* cells collected after centrifugation. The red and orange arrows indicate lycopene and β-carotene, respectively. The orange and yellow boxes represent chromatographic peaks extracted under the condition of characteristic ions of α-carotene and γ-carotene. The peak time is numerically marked. ‘N/A’ indicates no detection. **B** Contents of lycopene, γ-carotene, α-carotene, and β-carotene of the carotenoid extraction from *E. coli*. pACCRT-EIB, EIB. **C** Composition and proportions of the carotenoid extraction from *E. coli*. **D** Schematic diagram of the catalytic reactions of carrot DcLcyB proteins. The green, blue and orange lines represent reactions catalyzed by DcLcyE, DcLcyB1 and DcLcyB2, respectively

By heterologous expression in *Escherichia coli*, we analyzed the catalytic activity of DcLcyB proteins (Fig. 2). As a prerequisite, the introduction of the pACCRT-EIB plasmid led to the accumulation of lycopene in *E. coli*. After the co-expression of DcLcyB1 or DcLcyB2, the color of the bacteria shifted from red to yellow. The chromatogram displayed the significant peaks representing β-carotene, indicating that large amounts of β-carotene being produced (Fig. 2A). The contents of carotene produced by the co-expression of the two DcLcyB proteins were similar, being 10.42 and 10.90 μg/g, respectively (Fig. 2B). In addition, a small amount of α-carotene was detected in *E. coli* cells that co-expressed the pACCRT-EIB plasmid and the DcLcyB1 protein, which was absent in the group ‘pACCRT-EIB + DcLcyB2’. Conversely, the co-expression of DcLcyB2 resulted in the accumulation of γ-carotene, a monocyclic carotene. Although a small amount of γ-carotene was also accumulated in the cells expressing pACCRT-EIB plasmid alone, the amount was less than half of the group ‘pACCRT-EIB + DcLcyB2’. In this combination, the remaining amount of the lycopene substrate was high, accounting for 38% of all carotenoids (Fig. 2C). Overall, the total amount of carotenoids in *E. coli* expressing the DcLcyB1 alone and pACCRT-EIB plasmid was lower compared to other combinations, while the levels of other strains were comparable (Figure S3).

The formation of α-carotene typically demands the combined action of two lycopene cyclases, LcyE and LcyB. To further compare the contribution of DcLcyB1 and DcLcyB2 in this reaction, we also introduced the DcLcyE protein into *E. coli*. There were still 3 types of carotenes detected: α-carotene, β-carotene, and γ-carotene (Fig. 2A). Compared with the expression of the single DcLcyB protein, the β-carotene content in *E. coli* cells co-expressing DcLcyE increased by 56.54% and 45.04%, respectively. Similar to the combination pACCRT-EIB and DcLcyB1, *E. coli* co-expressing pACCRT-EIB, DcLcyE, and DcLycB1 accumulated β-carotene and a small amount of α-carotene, which accounted for 3% of the total carotenoid content (Fig. 2C). A surge of α-carotene was found in the cells co-expressing pACCRT-EIB, DcLcyE, and DcLcyB2. The accumulation of γ-carotene was still observed, but the content was quite low (Fig. 2B).

In general, DcLcyB1 was capable of cyclizing lycopene into two dicyclocarotenes, α-carotene and β-carotene. DcLcyB2 had a greater tendency to finish one-end cyclization, converting lycopene into γ-carotene or catalyzing the cyclization of monocyclic (δ-caortene or γ-carotene) to dicyclocarotene (Fig. 2D). It could also be said that the catalytic rate of DcLcyB2 in the cyclization reaction might be slower than that of DcLcyB1.

### Overexpression of *DcLcyB1/2* promoted downward xanthophyll metabolic flow

**Fig. 3:**
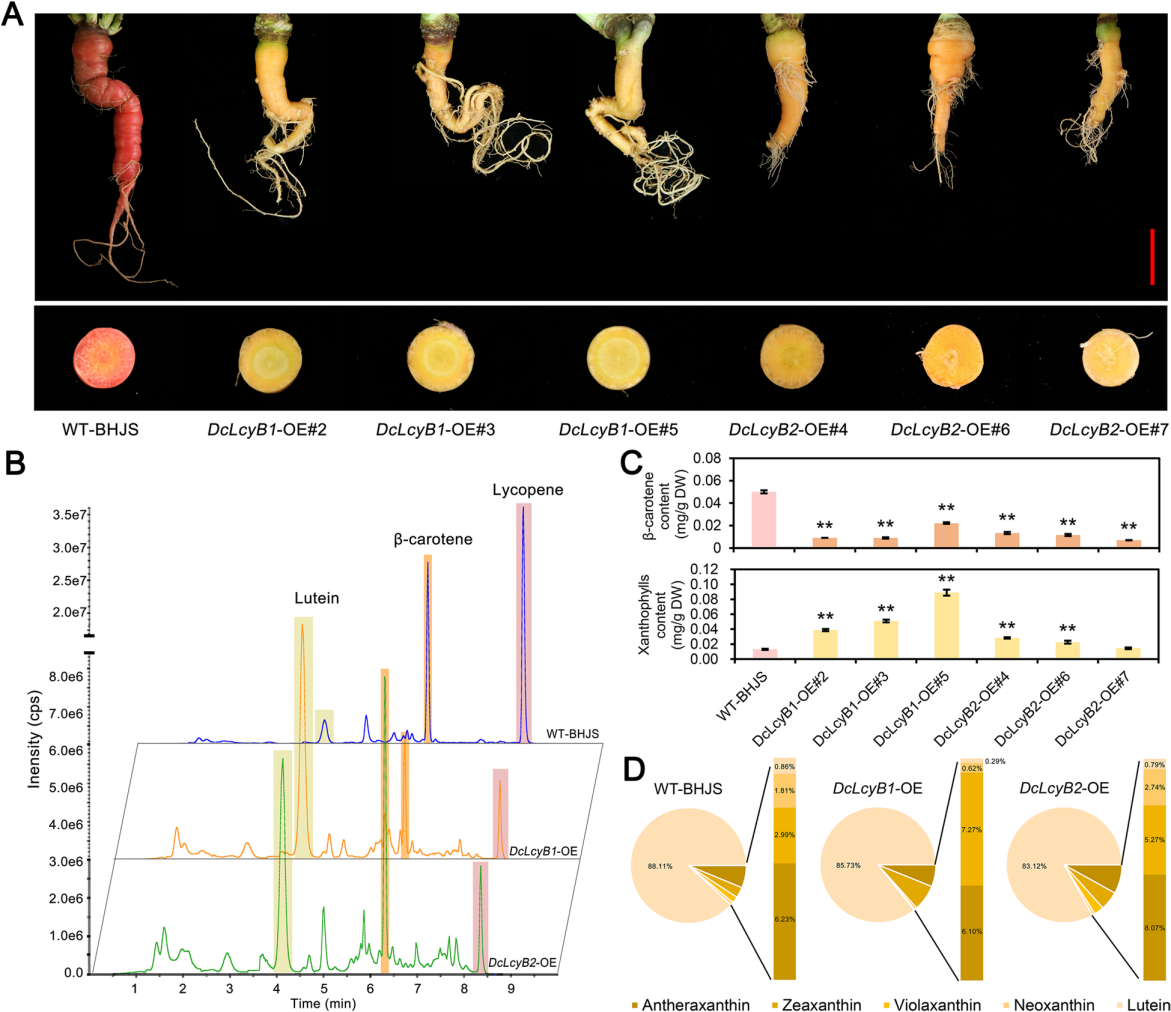
Overexpression of *DcLcyB1* or *DcLcyB2* gene in carrot BHJS. **A** Root and cross-sectional phenotypes of WT-BHJS, *DcLcyB1*-OE, and *DcLcyB2*-OE line carrot seedlings. The red line at the bottom right represents a 5-cm scale. **B** Total ion chromatography of carotenoid extraction from roots of WT-BHJS, *DcLcyB1*-OE, and *DcLcyB2*-OE line carrot seedlings by LC–MS/MS. The peaks representing lutein, β-carotene and lycopene are labeled with columns of different colors, respectively. **C** The contents of β-carotene and xanthophylls in roots of WT-BHJS, *DcLcyB1*-OE, and *DcLcyB2*-OE lines. ‘*’ and ‘**’ represent significant differences in *p* < 0.05 or *p* < 0.01 levels between transgenic lines and WT, respectively. Dry weight, DW. **D** The proportions of major xanthophylls, including antheraxanthin, zeaxanthin, violaxanthin, neoxanthin, and lutein in roots of WT-BHJS, *DcLcyB1*-OE, and *DcLcyB2*-OE line carrot seedlings

To precisely identify the function of DcLcyB proteins in carrot, *DcLcyB1* and *DcLcyB2* were separately overexpressed in carrot ‘Benhongjinshi’ (BHJS) (Fig. 3). The fleshy root of BHJS carrot accumulates a large amount of lycopene and do not contain α-carotene, facilitating the observation of the catalytic effect of DcLcyB protein on lycopene. From the obtained *DcLcyB* overexpression (*DcLcyB*-OE) positive plants, 3 lines were selected for subsequent functional analysis, namely *DcLcyB1*-OE#2, *DcLcyB1*-OE#3, *DcLcyB1*-OE#5 and *DcLcyB2*-OE#4, *DcLcyB2*-OE#6, *DcLcyB2*-OE#7, respectively. Each line contained 3 positive seedlings, as depicted in Fig. 3A. Unlike the bright red root of WT-BHJS carrot (control), the root of *DcLcyB1*-OE carrots appeared yellow, while *DcLcyB2*-OE tended to be orange-yellow.

Qualitative and quantitative analysis of carotenoids indicated that the main colored carotenoids in wild type (WT) carrot BHJS were lycopene, β-carotene, and lutein. After overexpression of *DcLcyB*, the content of lycopene decreased to a trace level (Fig. 3B). Interestingly, overexpression of *DcLcyB* did not lead to significant accumulation of β-carotene. On the contrary, the β-carotene in the fleshy roots of *DcLcyB1*-OE and *DcLcyB2*-OE lines decreased to 0.18 ~ 0.44 and 0.14 ~ 0.27 times of the WT-BHJS, respectively. Corresponding to the fleshy root color phenotype was the content of xanthophylls, overexpression of *DcLcyB1* or *DcLcyB2* resulted in significant increments in xanthophyll accumulations. The content of xanthophyll in *DcLcyB1*-OE lines was considerably higher than that in *DcLcyB2*-OE lines (Fig. 3C). Although the total amount of carotenoids in the *DcLcyB1*-OE lines was higher than that in the *DcLcyB2*-OE lines, the total carotenoid contents of both were significantly lower compared to that of the WT-BHJS (Figure S4). We further compared the content and proportion of 5 main xanthophylls, including antheraxanthin, zeaxanthin, violaxanthin, neoxanthin, and lutein (Fig. 3D, Table S2). The content of lutein was the highest, with its proportion ranging from 83.12% to 88.11% in WT and overexpression lines. Compared with the WT-BHJS and *DcLcyB1*-OE lines, the content of various lutein esters remained at relatively high levels in *DcLcyB2*-OE lines, such as lutein oleate, lutein palmitate, lutein dimyristate and lutein dipalmitate. Higher accumulation proportions of antheraxanthin in *DcLcyB2*-OE (8.07%) and zeaxanthin in *DcLcyB1*-OE (7.27%) were also detected.

### Co-expression of carotenoid pathway genes in *DcLcyB*-OE lines

**Fig. 4:**
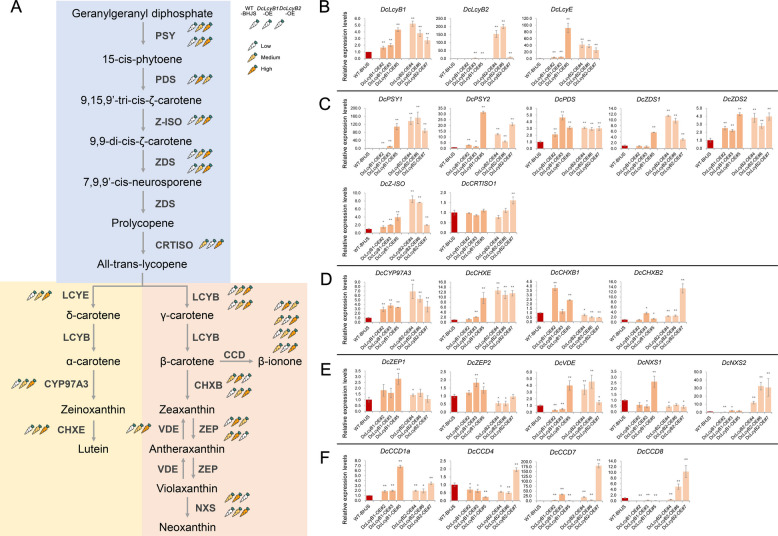
Relative expression levels of carotenoid pathway genes in roots of WT-BHJS, *DcLcyB1*-OE, and *DcLcyB2*-OE line carrot seedlings. **A** Carotenoid metabolic pathway in carrot. The different carrot symbols represent the expression levels of carotenoid pathway genes in WT-BHJS, *DcLcyB1*-OE and *DcLcyB2*-OE carrots. The gene expression levels were compared based on the average values of different transgenic lines, with high, medium and low expression represented by orange, yellow and white, respectively. In the context of multi-copy genes, each row corresponds to an individual gene. PSY, phytoene synthase; PDS, phyteoene desaturase; Z-ISO, 15-*cis*-ζ-carotene isomerase; ZDS, ζ-carotene desaturase; CRTISO, carotenoid isomerase; LcyE, lycopene ε-cyclase; LcyB, lycopene β-cyclase; CHXE, ε-ring carotene hydroxylase; CHXB, β-ring carotene hydroxylase; CYP97A3, cytochrome P450 carotene β-hydroxylase; CCD, carotenoid cleavage dioxygenases; VDE, violaxanthin de-epoxidase; ZEP, zeaxanthin epoxidase; NXS, neoxanthin synthase. **B** Relative expression levels of lycopene cyclase genes. **C** Relative expression levels of lycopene synthesis genes. **D** Relative expression levels of carotene hydroxylase genes. **E** Relative expression levels of xanthophyll synthesis genes. **F** Relative expression levels of carotenoid cleavage dioxygenase genes. ‘*’ and ‘**’ represent significant differences in *p* < 0.05 or *p* < 0.01 levels between transgenic lines and WT, respectively

To understand the effect of the *DcLcyB1/2* gene on the entire carotenoid metabolic pathway, the relative expression levels of pathway genes were measured by RT-qPCR (Fig. 4A). The expression levels of the *DcLcyB1* gene in *DcLcyB1*-OE transgenic carrot lines were approximately 2 to 4 times than those of WT-BHJS. The expression level of *DcLcyB2* in the overexpression lines increased significantly, even reaching over 100 times in lines *DcLcyB2*-OE#4 and *DcLcyB2*-OE#6. In the three *DcLcyB1*-OE lines, the expression level of *DcLcyB2* increased slightly, averaging approximately twice that of the WT-BHJS. More obviously, the expression level of *DcLcyB1* in *DcLcyB2*-OE lines was elevated to 5.24, 3.81, and 2.74 times that of WT-BHJS, respectively. There was also a surge in the *DcLcyE* expression levels, especially in the *DcLcyB2*-OE lines, reaching 41.97 times (OE#4) (Fig. 4B). The expression levels of almost all genes related to linear lycopene synthesis were significantly increased, among which *DcPSY1* (152.64 folds in *DcLcyB2*-OE#6 than WT-BHJS) and *DcPSY2* (21.02 folds in *DcLcyB2*-OE#7 than WT-BHJS) were the most prominent. The remaining genes such as *DcZDS1* and *DcZ-ISO* also exhibited a nearly tenfold rise. In general, the expressions of these genes were higher in *DcLcyB2*-OE lines than in *DcLcyB1*-OE lines (Fig. 4C). Overexpression of *DcLcyB* also enhanced the expression of carotene hydroxylase genes, especially in the *DcLcyB2*-OE lines. In particular, the expression of the *DcCHXB1* gene in *DcLcyB2*-OE lines was inhibited, being only 0.48 times (*DcLcyB2*-OE#7) that in WT-BHJS (Fig. 4D). Both zeaxanthin epoxide genes, *DcZEP1* and *DcZEP2*, expressed at higher levels in the *DcLcyB1*-OE lines. The expression levels of the two *DcNXS* genes were opposite in the two *DcLcyB*-OE lines, with *DcNXS1* being higher in the *DcLcyB1*-OE lines and *DcNXS2* being higher in the *DcLcyB2*-OE lines (Fig. 4E). We also determined the expression levels of four *DcCCD* genes in transgenic carrot lines (Fig. 4F). The expression levels of *DcCCD1a* and *DcCCD7* in *DcLcyB1* and *DcLcyB2* overexpressing lines were all increased compared to the WT-BHJS, which was most significant in *DcLcyB1*-OE#5 and *DcLcyB2*-OE#7 lines. *DcCCD4* expressed significantly lower than WT-BHJS in most overexpression lines. *DcCCD8* exhibited a lower expression level in the *DcLcyB1* overexpression lines relative to WT. Conversely, in *DcLcyB2*-OE#6 and *DcLcyB2*-OE#7 lines, the expression levels of *DcCCD8* were elevated to 5.03- and 10.33-fold of that in the WT-BHJS, respectively.

Given that the expression of most carotenoid pathway genes was collectively increased in *DcLcyB* overexpressing plants, we hypothesized that interactions might exist among them. As shown in Figure S5, DcLcyB1 may interact with several carotenoid biosynthesis pathway proteins, including multiple carotene hydroxylases CHXB (BCH), CYP97A3, and CHXE, ζ-carotene desaturase (ZDS), and carotenoid isomerase (CRTISO). In addition, two DWARF proteins encoding β-carotene isomerase were also identified. All the identified interacting proteins were associated with the carotenoid pathway. As for DcLcyB2 (Figure S6), in addition to BCH1/2, DcLcyB2 may also interacted with zeaxanthin epoxidase (ZEP) and 9-*cis*-epoxycarotenoid dioxygenase (CCD4), and two isomerases (Z-ISO and CRTISO). MTS (2-C-methyl-D-erythritol 2,4-cyclodiphosphate synthase) is a key enzyme in the MEP (methylerythritol phosphate) pathway for the synthesis of carotenoid substrate (Kumar et al 2012). NDC80 (kinetochore protein Ndc80) is a crucial component of kinetochore complex (Muir et al 2023). KAR (ketol-acid reductoisomerase) is mainly involved in amino acid biosynthesis (Yu et al 2020).

### Knocking out of DcLcyB protein changed the α-/β-carotene ratio

**Fig. 5:**
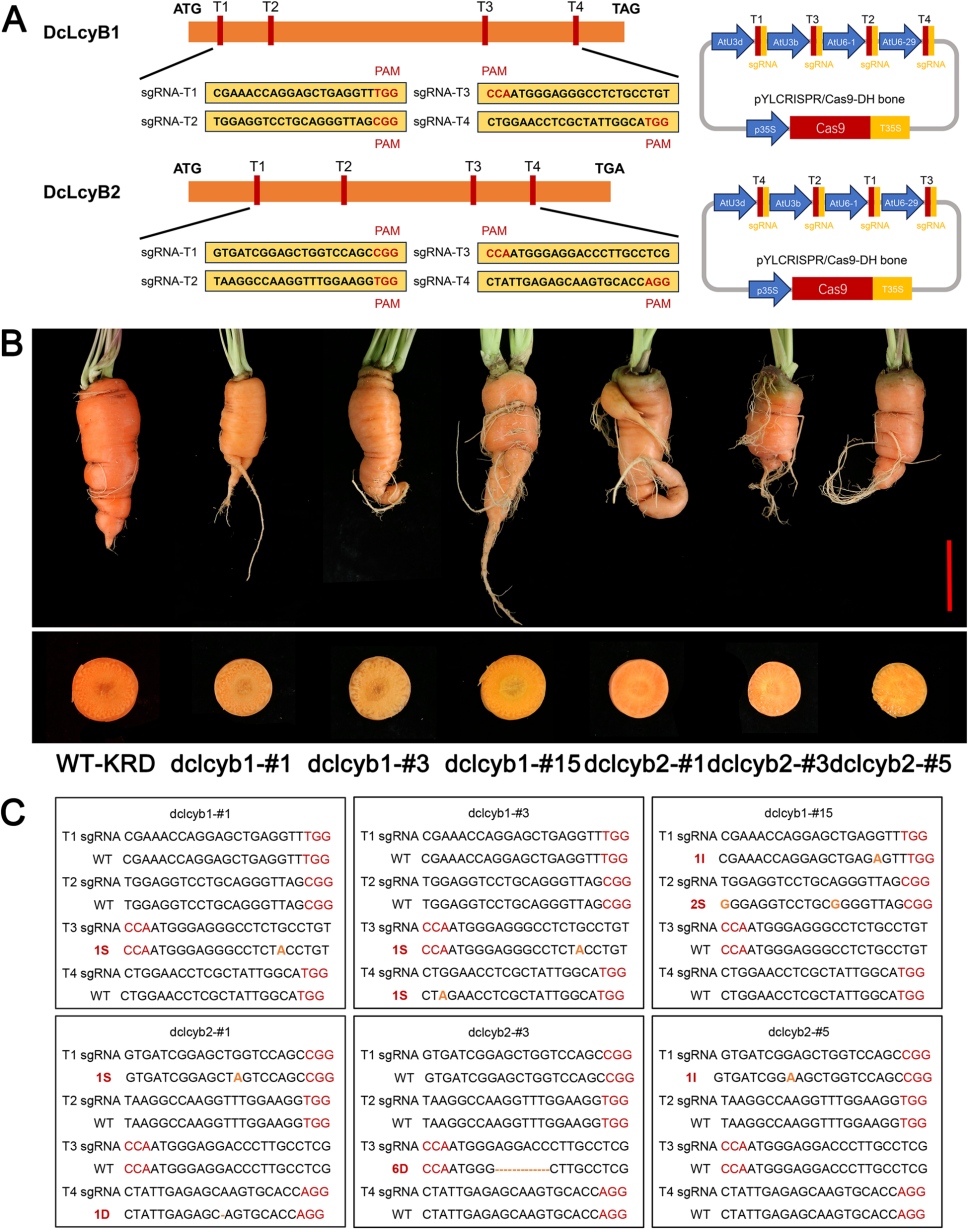
Gene editing of *DcLcyB1* and *DcLcyB2* in carrot KRD. **A** Target sites and vector construction of *DcLcyB1* and *DcLcyB2* gene editing. T1 ~ T4 represent target sites 1 to 4. PAM, protospacer adjacent motif; p35S, CaMV*35S* promoter; T35S, CaMV*35S* terminator. **B** Root and cross-sectional phenotypes of WT-KRD, *dclcyb1*, and *dclcyb2* mutants. The red line at the bottom right represents a 5 cm scale. **C** Identification of target site sequence of *dclcyb1* and *dclcyb2* mutants. S, I, and D represent base substitution, insertion, and deletion, respectively. The number before the letter represents the number of mutant bases. The mutant sites are marked in orange

Based on the CRISPR/Cas9 system, gene knockout of *DcLcyB1* and *DcLcyB2* were conducted in carrot KRD. KRD is a type of orange-root carrot, with a high content of both α-carotene and β-carotene, and the functional verification of DcLcyBs in this cultivar can readily exhibit the changes of the α- and β-branches. Four target sites were chosen for each gene, and the location and other relevant information of the target sites were presented in Fig. 5A and Table S3. Each target site was linked with a specific promoter and subsequently connected to the pYLCRISPR/Cas9-DH vector backbone in a certain sequence. As depicted in Fig. 5B, after gene editing, the root color of the carrot mutant became paler and turned orange-yellow. The sequence identification of the target sites indicated that the *dclcyb* mutants had variations such as substitution (*dclcyb1*-#1, #3, and #15; *dclcyb2*-#1), insertion (*dclcyb1*-#15; *dclcyb2*-#5), and deletion (*dclcyb2*-#1 and #3) (Fig. 5C). Table S4 illustrated the impacts of gene editing sites on the amino acid residues of the DcLcyB proteins. These variations resulted in alterations to the amino acid residues, as well as the occurrence of premature termination or frameshift mutations of DcLcyB1 and DcLcyB2 proteins, ultimately impairing the protein function. For the *dclcyb1*-#1 and *dclcyb1*-#3, although only single-base synonymous mutations were detected at the target sites, a large number of amino acid frameshift mutations occurred downstream of the target site.

**Fig. 6:**
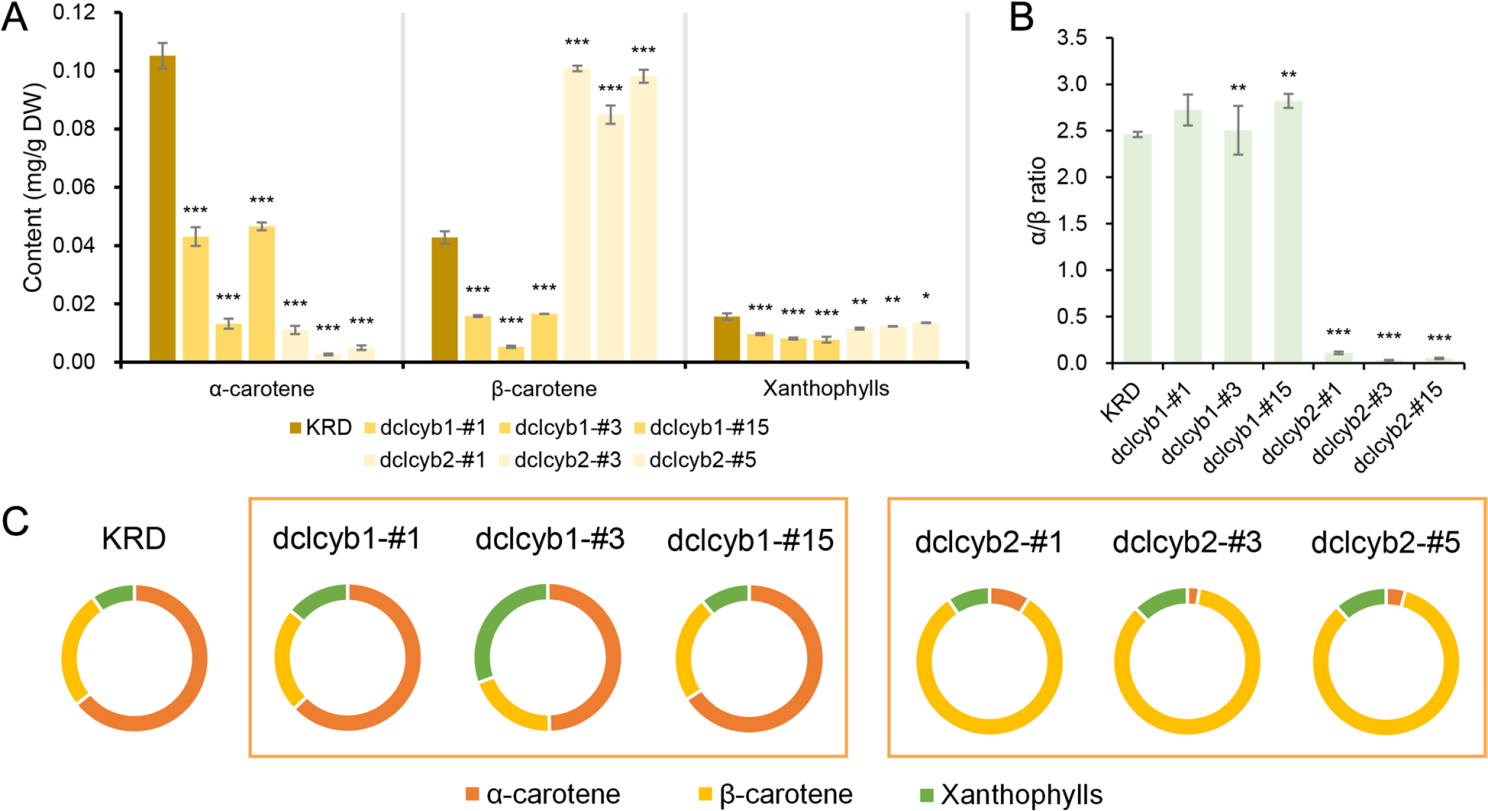
Carotenoid accumulation in roots of WT-KRD, *dclcyb1*, and *dclcyb2* carrot mutants. **A** Contents of α-carotene, β-carotene, and xanthophylls in roots of WT-KRD, *dclcyb1*, and *dclcyb2* mutants. ‘*’ and ‘**’ represent significant differences in *p* < 0.05 or *p* < 0.01 levels between transgenic lines and WT, respectively. **B** The ratio of α-carotene to β-carotene in roots of WT-KRD, *dclcyb1*, and *dclcyb2* mutants. ‘*’ and ‘**’ represent significant differences in *p* < 0.05 or *p* < 0.01 levels between transgenic lines and WT, respectively. **C** Composition and proportion of carotenoids in roots of WT-KRD, *dclcyb1*, and *dclcyb2* mutants. Total carotenoids were represented by the sum of α-carotene, β-carotene, and xanthophylls

The pattern of carotenoid accumulation in WT-KRD and *dclcyb1/2* mutants were compared. The results indicated that the α-carotene content in *dclcyb* mutants decreased significantly, particularly in the *dclcyb2* mutant, with an average of only 0.17 times that of WT-KRD. The accumulation of β-carotene was inhibited in the *dclcyb1* mutant; however, surprisingly, β-carotene levels in *dclcyb2* remained high, even exceeding those of the wild type. The content of xanthophylls also decreased significantly, being 0.49 to 0.87 times that of the WT-KRD. The content in WT-KRD was 0.016 mg/g dry weight (DW), while in the *dclcyb1* and *dclcyb2* mutants, it ranged from 0.008 to 0.009 mg/g DW and 0.012 to 0.013 mg/g DW, respectively (Fig. 6A). After knocking out the two *DcLcyB* genes respectively in the carrot KRD, the total carotenoid content decreased significantly. In comparison, the total amount of carotenoids in the *dclycb2* mutants was higher than that in the *dclcyb1* mutants (Figure S7). Using LC–MS/MS, precise qualitative and quantitative analysis of different types of xanthophylls were conducted. The contents of antheraxanthin, zeaxanthin, violaxanthin, neoxanthin, and lutein are shown in Figure S8. In the *dclcyb1* mutants, the accumulation of antheraxanthin, violaxanthin, and neoxanthin was less than WT-KRD. Similarly, the *dclcyb2* mutant exhibited the same trend, but the decrease in content was relatively small. The change of zeaxanthin was not obvious in the *dclcyb1*, while the content in the *dclcyb2* elevated. From the perspective of proportion, the α-/β-carotene ratio in the *dclcyb1* increased slightly, but decreased sharply in the *dclcyb2* (Fig. 6B). Overall, the proportion of α-carotene in the *dclcyb2* mutant decreased to a minimum of 3%, while it reached more than 60% in the WT-KRD (Fig. 6C).

### Response of carotenoid pathway genes to impaired DcLcyB protein function

**Fig. 7:**
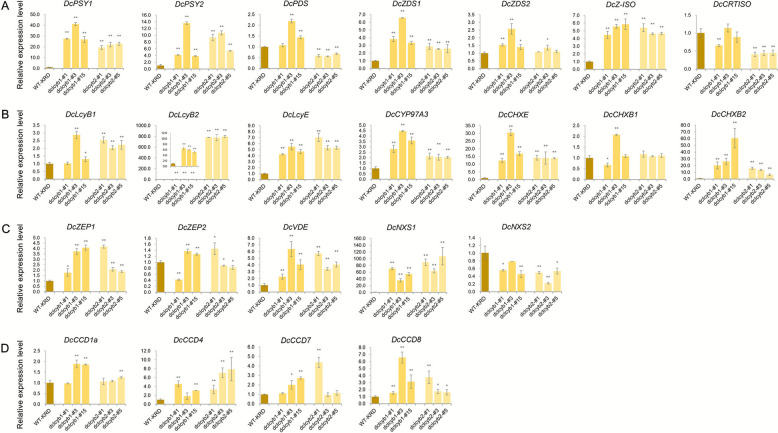
Relative expression levels of carotenoid pathway genes in roots of WT-KRD, *dclcyb1*, and *dclcyb2* carrot mutants. **A** Relative expression levels of lycopene synthesis related genes. **B** Relative expression levels of carotene-related genes. **C** Relative expression levels of xanthophyll cycle related genes. **D** Relative expression levels of carotenoid cleavage dioxygenase genes. ‘*’ and ‘**’ represent significant differences in *p* < 0.05 or *p* < 0.01 levels between transgenic lines and WT, respectively

In view of the substantial alteration of carotenoid content in *dclcyb1/2* carrot mutants, the expression levels of carotenoid biosynthetic pathway genes were determined using RT-qPCR (Fig. 7). The expression of almost all genes (*DcPSY1*, *DcPSY2*, *DcZDS1*, *DcZDS2*, and *DcZ-ISO*) in the synthesis stage from carotenoid substrate geranylgeranyl diphosphate (GGPP) to lycopene was increased in the roots of *dclcyb1* and *dclcyb2* mutants (Fig. 7A). The three lycopene cyclase genes *DcLcyB1*, *DcLcyB2*, along with *DcLcyE* were expressed significantly higher than those of WT. In particular, the expression of *DcLcyB2* in *dclcyb2* mutants reached a level of hundreds of times. The carotene hydroxylase genes were also facilitated, and the expression levels of *DcCHXE* and *DcCHXB2* in the two *dclcyb* mutants reached tens of times that of WT-KRD (19.92 and 35.72 folds on average in *dclcyb1*; 14.02 and 12.05 folds on average in *dclcyb2*) (Fig. 7B). *DcZEP*, *DcVDE*, and *DcNXS* are genes that encodes enzymes associated with the xanthophyll cycle (Fig. 7C). In the two mutations of *DcLcyB* genes, the changing tendencies of these genes were similar. The expression level of *DcNXS1* was particularly up-regulated in the *dclcyb* mutants, which was 53.73 and 86.82 times that of WT, respectively. Although the expression levels of *DcCCD* genes (*DcCCD1a*, *DcCCD4*, *DcCCD7*, and *DcCCD8*) in some mutants did not differ significantly from those in the WT-KRD, the *DcCCD* expression levels generally exhibited upward trends both in the *dclcyb1* and *dclcyb2* mutants (Fig. 7D).

## Discussion

### *DcLcyB1* and *DcLcyB2* encodes vital cyclase required for carrot carotenoid accumulation

Lycopene cyclase in plants can be divided into LcyE and LcyB based on the disparity of cyclization function (Giuliano 2014; Nisar et al 2015). Most species possess one LcyE and one LcyB, respectively, while in a few species, a second LcyB exists, frequently referred to as LcyB2 or CCS (Guzman et al 2010; Jeknic et al 2012; Mialoundama et al 2010). Although the carotenoid pathway proteins including LcyB are typically plastid-targeted proteins, DcLcyB2/DcCCS is regarded as chromoplast-specific proteins, and is commonly found in fruits and other organs rich in pigmentation (Wang et al 2019; Rosas-Saavedra et al 2023). The fleshy roots of carrots are a typical natural tissue abundant in carotenoids, which are stored in chromoplast (Zhang et al 2022). Studies have proved that the fleshy roots of early wild carrots were non-pigmented, and as the species evolved, the roots transformed into purple, yellow, orange, or red colors (Coe et al 2021; Wang et al 2024), to which *LcyB* genes might have contributed in this evolutionary process. According to the current and previous studies (Rosas-Saavedra et al 2023; Wang et al 2023a), the transcriptional level of *DcLcyB2* in carrot roots was relatively higher than *DcLcyB1*, confirming its possible predominant role in carotenoid accumulation. However, an alternative possibility is that DcLcyB2 protein exhibited relatively low enzymatic activity, necessitating a higher transcriptional level to compensate for this deficiency.

Significant differences in the extent of transcriptional upregulation between *DcLcyB1* and *DcLcyB2* in their respective overexpression lines were observed in our study. When the same promoter was used to drive their overexpression in carrot, the expression level of *DcLcyB2* showed a far more dramatic increase (Fig. 4B). This likely reflected differences in the transcriptional efficiency of these two *DcLcyB* genes. Specifically, the promoter region of *DcLcyB2* may contain more *cis*-acting elements that facilitate RNA polymerase binding, enabling more efficient transcription initiation under the *35S* promoter and thus producing a greater number of transcripts (Butler & Kadonaga 2002). Additionally, the two genes may differ in mRNA stability and translation efficiency. Differences in their genomic integration sites might also contribute to this phenomenon. The chromatin state could inhibit the expression of transgenes through histone modifications and DNA methylation (Kohli et al 2003). *DcLcyB2* may have integrated into a more transcriptionally active genomic region. Future research on the post-transcriptional regulatory mechanisms of the *DcLcyB* genes may lead to more valuable discoveries.

The cyclization process of LcyB, a crucial branch of carotenoid synthesis, can directly result in the color conversion of carotenoid. The abundance of ClLCYB protein can regulate the transformation of white, red and yellow flesh in watermelon (Alquezar et al 2009; Zhang et al 2020). In carrots, the gene governing the red root phenotype at the key locus does not seem to be encoded by the lycopene cyclase gene. In our previous study, the knockout of *DcLcyE* did not cause lycopene accumulation in carrot roots (Wang et al 2023c), nor did the editing of *DcLcyB1/2* in this study. Recent study has detected the production of lycopene in carrot callus through gene editing of lycopene cyclase genes, but no evidence was provided to verify that lycopene could be retained in the differentiated carrot roots (Li et al 2025). Although Jo et al (2021) discovered an insertion in the amino acid of DcLCYB2 protein in one red carrot source, which could affect the catalytic activity of DcLCYB2, this does not appear to be the cause of red carrot roots. According to the sequence results of DcLcyB protein cloned from different carrot cultivars, no amino acid mutations related to color were identified (Figure S2). Therefore, the origin of carrot red root is not a simple mutation of structural gene, but may involve multiple factors such as the regulation of upstream transcription factors and the storage form of lycopene.

### Catalytic substrate bias of two lycopene β-cyclases

The major catalytic function of lycopene β-cyclase lies in the formation of the β-ring (Liang et al 2019). Cyclic carotenes can be classified into monocyclic carotene (γ-carotene and δ-carotene) and dicyclocarotene (α-carotene, β-carotene, and ɛ-carotene) according to the cyclic structures (Cunningham et al 1996). In this study, both two DcLcyB proteins catalyzed the lycopene substrate into a large amount of β-carotene, while in the by-product, the catalytic reaction of DcLcyB1 contained a small quantity of α-carotene, and that of DcLcyB2 was γ-carotene, and the remaining amount of lycopene substrate was also higher (Fig. 2). This result indicated that the two DcLcyB proteins may have difference in their affinity for substrates and catalytic efficiency. DcLcyB1 could rapidly catalyze the conversion of lycopene to β-carotene even when the concentration of lycopene in *E. coli* was relatively low. As lycopene continued to be produced, it was continuously consumed by DcLcyB1. In contrast, DcLcyB2 had weaker binding affinity for lycopene. It catalyzed the reaction efficiently only when substrate concentrations were high. Furthermore, its catalytic efficiency was also low, indicating that it converted less substrate per unit time. This means that the rate at which DcLcyB2 consumed lycopene could not keep pace with the rate at which lycopene was produced, leading to residual lycopene. We also hypothesized that DcLcyB2 might have a stronger preference for monocyclic carotene substrates. DcLcyB2 prefers the seemingly simple reaction that catalyzing only single-end cyclization in a single step rather than the sequential cyclization of lycopene at both ends.

The special α-carotene possesses an ɛ-ring and a β-ring at each end, thereby necessitating the combined action of the two cyclases (Wang et al 2022a, b). In combination with the previous study on the function of DcLcyE (Wang et al 2023c), we introduced the DcLcyE protein into the reaction to further explore the coordination of the two important roles of DcLcyE and DcLcyB in this catalytic process. DcLcyE contributed the monocyclic carotene substrate to the reaction system. In plant, LcyE usually co-exists with LcyB (Koc et al 2015), and this reaction system can better reflect the mechanism of carotene accumulation in plants. Consistent with our hypothesis, these monocyclic carotene substrates seem to be favored by DcLcyB2, leading it to produce a lot of α-carotene. Validation in carrots could assist us in better understanding the function of DcLcyB. In the roots of *dclcyb2* carrot mutants, the content of α-carotene along with the α-/β-carotene ratio was significantly reduced, and the extent of decline was significantly higher than that of *dclcyb1*. This result demonstrated the contribution of DcLcyB2 in enriching the diversity of carotenes, especially α-carotene.

### Indirect regulation of xanthophyll synthesis by lycopene β-cyclases

In plants, the principal function of LcyB is to catalyze the synthesis of β-carotene. This conclusion has been verified in numerous crops, overexpression of *LcyB* can cause a significant increase in β-carotene content (Diretto et al 2020; Kang et al 2018; Zhao et al 2022a, b). Previous studies have indicated that transgenic carrot plants with higher levels of *DcLcyB1* had incremented levels of total carotenoids and β-carotene (Moreno et al 2013). Overexpression of *DcLcyB2* in *N. tabacum* led to an increase in the levels of β-carotene, lutein, and chlorophyll (Rosas-Saavedra et al 2023). Unexpectedly, we failed to detect the anticipated high levels of β-carotene in carrot *DcLcyB*-OE lines, but a remarkable change in xanthophylls was observed. We speculated that this phenomenon might be related to the background carrot cultivar we chose. Compared with *E. coli* strains engineered to harbor exogenous carotenoid metabolic pathways, the host environment of carrots is far more complex and also species-specific. After overexpressing the *DcLcyB* gene in carrots, the endogenous feedback mechanisms might be activated (Cazzonelli & Pogson 2010), the distribution of substrates among different metabolic branches might be altered (Wang et al 2023c), and the synergistic or antagonistic effects of other genes associated with carotenoid biosynthesis would further disrupt the original metabolic homeostasis (Wang et al 2024). The transcriptional level changes of numerous carotenoid pathway genes observed in the transgenic carrots we observed could support this view. In this study, red root carrot BHJS was chosen as the material for overexpression in order to provide sufficient lycopene substrate. The transcriptional regulation of carotenogenesis in BHJS differs from that of orange carrot (Wang et al 2020). Upon overexpression of both *DcLcyB1* and *DcLcyB2*, the carotenoid metabolism in the roots of BHJS carrot was transformed towards the downstream xanthophylls, especially lutein and zeaxanthin. The result of carotenoid pathway gene expression indicated that the carotene hydroxylase genes (*DcCHXE*, *DcCYP97A3*, *DcCHXB1* and *DcCHXB2*) exhibited a co-expression trend. The carotenoid hydroxylase gene in BHJS already exhibits a relatively high expression level rather than orange carrots (Wang et al 2020), which was further enhanced after overexpression of *DcLcyB*. Studies on tomato have also shown that overexpression of *LCYB* or *CCS* genes in *E. coli* or tomatoes respectively increased the content of xanthophylls and xanthophyll esters, demonstrating the promoting effect of *LcyB* on the accumulation of xanthophylls (Fu et al 2024). The prediction of protein interaction also suggested that there might be protein interaction between DcLcyB proteins and carotene hydroxylase protein, as well as other enzymes in the carotenoid pathway. We attempted to verify the interactions among different carotenoid pathway genes using yeast two-hybrid technology both in this study and our other work (Deng et al 2025), but failed to observe the interactions. This suggested that the regulation of genes in carotenoid pathway may be not occur solely through direct protein–protein interactions; instead, the overall balance of the entire metabolic pathway should be considered. The results also highlight when engineering carotenoid biosynthesis via* LcyB* gene in future studies, the background effects of the host organism must be considered.

### Dynamic change and balance of carotenoid metabolic pathway

There is a delicate equilibrium within the entire carotenoid metabolic pathway, and this balance can be affected by the precursors and intermediates, as well as by the various catalytic enzymes on the pathway (Bai et al 2016). Upon the occurrence of change in one structural gene, the genes throughout the pathway tend to exhibit a coordinated response.

Our results revealed that with overexpression of the *DcLcyB* genes, the carotenoid metabolism pathway did not simply lead to the accumulation of β-carotene and then come to a halt. Instead, it simultaneously boosted the flux of downstream reactions, which in turn gave rise to an increase in the content of xanthophylls, especially the proportion of zeaxanthin, the first product of the conversion of β-carotene to xanthophyll. Correspondingly, the expression levels of carotenoid hydroxylase genes were elevated to varying extents in the *DcLcyB*-OE plants (Fig. 4). Both *DcCYP97A3* and *DcCHXE* were genes encoding carotenoid hydroxylases that are primarily responsible for the conversion of α-carotene to lutein (Deng et al 2025; Wang et al 2025). In comparison with *DcLcyB1*-OE lines, the expression levels of *DcCYP97A3* and *DcCHXE* in *DcLcyB2*-OE lines exhibited a more pronounced increase, which might be associated with the more significant involvement of DcLcyB2 protein in the α-carotene branch. The downstream pathways promoted by DcLcyB were not limited to carotenoid biosynthesis, they also extended to carotenoid degradation. The expression levels of *DcCCD* genes exhibited a responsive change accordingly. In return, for the genes encoding the enzymes that catalyze the synthesis of lycopene in the upstream process (*DcPSYs*, *DcPDS* and *DcZDSs*), higher expression was required to provide sufficient lycopene for the subsequent metabolic flow.

This response was also observed in *dclcyb* mutants, where the non-functionality of one enzyme led to the upregulation of the expression levels of other enzymes in the pathway, thereby maintaining the normal flow of the total metabolic process. In *dclcyb* knockout mutants, since both two DcLcyB proteins possess β-cyclization activity, when one of them was knocked out, the other may exert a compensatory effect. For the two DcLcyB proteins, they may differ in catalytic functions, but were generally redundant to some extent. Specifically, when the function of one DcLcyB protein was absent, the transcription level of the other isoenzyme was significantly increased, thus achieving the replenishment of enzyme activity (Fig. 7B). In *dclcyb2* mutants, the deficiency of DcLcyB2 resulted in reduced α-carotene content; however, the concurrent upregulation of *DcLcyB1* expression maintained the biosynthesis of β-carotene even beyond those in WT plants (Fig. 6A). For downstream hydroxylase genes, their expression was coordinately and indirectly activated alongside the *DcLcyB1* gene. Nevertheless, a reduction in the content of xanthophylls on the β-carotene branch (antheraxanthin, violaxanthin, and neoxanthin), accompanied by an increase in lutein levels could be observed in *dclcyb* mutants (Figure S6), reflecting the influence of the DcLcyB proteins on the two branches of carotenoids. This regulatory coordination not only preserved the flux balance of the carotenoid biosynthetic pathway but also maintained cellular redox homeostasis. Overall, whether the *DcLcyB* gene was overexpressed of knocked out, the phenotypic performance of the resulting transgenic carrot was a consequence of the coordinated response of the entire carotenoid biosynthetic pathway, rather than being solely by the alteration of a single gene or a single catalytic reaction.

In summary, the current study elucidated the functional disparity of two DcLcyB proteins in carrot and their regulatory mechanisms concerning the carrot fleshy root color. Beyond the impact on the synthesis of β-carotene by DcLcyB, the results also suggested that DcLcyB2 might possess a stronger substrate preference for monocyclic carotene during cyclization, thus facilitating the synthesis of α-carotene. The synergistic regulation of DcLcyB protein on xanthophyll accumulation was also verified. These findings can offer insights into the targeted manipulation of carotenoid accumulation in carrot and other plants.

## Materials and methods

### Plant materials and growth conditions

Orange root carrot ‘KRD’ was used for gene cloning and vector construction. A total of 7 carrot varieties with distinct root colors were selected as experimental materials for gene expression analysis, including ‘Diwang’ (DW, orange), ‘Leikende’ (LKD, orange), ‘Meiguihong’ (MGH, red), ‘Qitouhuang’ (QTH, yellow), ‘Deep purple’ (DPP, purple), ‘Baiyu’ (BY, white), and ‘Nanjingyesheng’ (NJYS, white). All carrot seeds were stored in the State Key Laboratory of Crop Genetics & Germplasm Enhancement and Utilization, Nanjing Agricultural University (Nanjing, China). After a short period of accelerated germination, the well-germinated seeds were selected and transplanted into the culture soil and grew in the greenhouse. At 90 days after sowing (DAS), the samples were collected from the aboveground part (including leaves and petioles) as well as the belowground part (roots). Each sample was taken from 5 different individual plants and mixed uniformly.

### Extraction of DNA, RNA and cDNA

The extraction of DNA and RNA was performed in accordance with the instructions of the kits. The Novel Plant Genomic DNA Extraction Kit (AG501C) and Polysaccharide Polyphenol Plant Total RNA Extraction Kit (AF502A) were both purchased from ProteinssciBiotech Company (Shanghai, China). RNA samples were reverse transcribed into cDNA using HiScript II Q RT SuperMix for qPCR (R223-01, Vazyme, Nanjing, China). The cDNA samples were diluted 18-fold for subsequent amplification.

### Identification of LcyB proteins

The sequences of the DcLcyB1 and DcLcyB2 proteins in carrot were obtained based on the *Daucus carota* vT2T genome (Wang et al 2023a). The secondary structure of DcLcyB proteins was predicted by SWISS-MODEL web service (Waterhouse et al 2018) and presented by ESPript 3.0. Lycopene cyclase proteins (including crtY, LcyB and LcyE) in other species were searched based on homology with the help of Phytozome v14 online database. The LcyB/E proteins of multiple species were aligned by the clustalW method and the phylogenetic tree was generated by the neighbor-joining method using MEGA-X (Kumar et al 2018). The protein tertiary structure of DcLcyB proteins was obtained from the AlphaFold Protein Structure Database (https://alphafold.ebi.ac.uk/) (Jumper et al 2021), and the binding site motif were annotated using the Discovery Studio 2025 Client software. The interacting proteins of DcLcyB proteins were predicted by STRING database (Szklarczyk et al 2023).

### Quantitative real-time PCR (RT-qPCR)

The relative expression levels of genes were analyzed by high-sensitivity quantitative SYBR dye PCR. The PCR amplification was conducted on the BioRad CFX96 platform using the ChamQ SYBR qPCR Master Mix premixed solution. The reaction system was first subjected to initial denaturation step at 90 ℃ for 30 s, followed by 40 cycles consisting of 95 ℃ for 10 s and annealing at 60 ℃ for 30 s. Cycle threshold values (Ct) were converted into relative expression levels using the 2^−△△Ct^ method (Pfaffl 2001). The *DcActin* gene was used as the reference gene (Wang et al 2016). The primer sequences used for RT-qPCR were detailed in Table S1.

### Gene expression and gene editing vector construction

For prokaryotic expression, the full coding regions of *DcLcyB1* and *DcLcyB2* were amplified the using primer sets *DcLcyB1*-pGEX-F/R and *DcLcyB2*-pGEX-F/R (Table S1). The resulting PCR products were introduced in to the *EcoR*I and *Xho*I restriction sites and then constructed onto the *E. coli* expression vector pGEX-5X-1.

For gene overexpression, gene *DcLcyB1* or *DcLcyB2* was inserted into the vector pCAMBIA1301, which was equipped with the *CaMV35S* promoter and the TPS terminator. The related primers used were listed in Table S1.

For gene editing, the pYLCRISPR/Cas9Pubi-H vector, which is appropriate for dicotyledonous plants, was selected following the previous method (Xu et al 2019). According to the genome sequences of *DcLcyB1* and *DcLcyB2*, target sites located upstream of the protospacer adjacent motif (PAM) sequence were screened with the help of the targetDesign online software (http://skl.scau.edu.cn/targetdesign/). Four target sites were chosen according to the location, GC content, specificity and other relevant parameters of the target sequence. The target site sequences were inserted into the backbone of the vector after being linked to four promoter *AtU3d*, *AtU3b*, *AtU6-1*, and *AtU6-29* along with the sgRNA, respectively.

### Heterologous expression in *E. coli*

Lycopene substrates were synthesized in *E. coli* utilizing the plasmid pACCRT-EIB containing the bacterial enzymes crtE, crtI, and crtB. The plasmid was developed and generously provided by Professor Norihiko Misawa from Ishikawa Prefectural University (Yamano et al 1994). The constructed DcLcyB1/2-pGEX plasmids were co-transformed with pACCRT-EIB into *E. coli* BL21 (DE3). To assess the substrate preferences of DcLcyBs, the DcLcyB1/2-pGEX plasmids were also co-transformed into *E. coli* alongside the previous constructed DcLcyE-PET30. The co-expression of DcLcyE-PET30 and pACCRT-EIB could facilitate the accumulation of monocyclic carotenes in *E. coli* (Wang et al 2023c). The transformed positive colonies were propagated in LB liquid medium supplemented with antibiotics and cultured under dark conditions.

### Genetic transformation in carrots

The constructs for plant overexpression and gene editing were transformed into *Agrobacterium tumefaciens* strain GV3101. Carrot ‘BHJS’ with lycopene accumulation was used as the material for overexpression of the *DcLcyB1* and *DcLcyB2* genes. *DcLcyB* gene editing lines were generated from the orange-rooted carrot ‘KRD’. The genetic transformation of carrots was conducted according to previously published methods (Wang et al 2023c). DNA was extracted from carrot seedlings for PCR to identify positive transformants, the cloning primers and vector specific primers were used for detection (Table S1). Enlarged carrot fleshy roots were harvested after 3 months of growing. Three individual plants from each *DcLcyB1/2* overexpression line were selected for biological replicates, and the *dclcyb1/2* gene editing samples were collected from each plant.

### Qualitative and quantitative analysis of carotenoids

The extraction and identification of carotenoids were performed by ultra-high performance liquid chromatography (UPLC) and liquid chromatograph-tandem mass spectrometer (LC–MS/MS). For UPLC, acetone was used for the repeated extraction of carotenoids from freeze-dried samples. The determination was carried out with acetonitrile and methanol as mobile phases according to previous methods (Wang et al 2023c). For the LC–MS/MS analysis, the ground samples were extracted with a solvent mixture of hexane, acetone, and ethanol (1:1:1, v/v/v) containing 0.01% butylated hydroxytoluene (BHT), followed by concentration and redissolution in dichloromethane. Chromatographic acquisition was performed using a YMC C_30_ column, with methanol/acetonitrile and methyl tert-butyl ether as the mobile phases A and B, respectively. Mass spectrometry data was collected at an atmospheric pressure chemical ionization source temperature of 350 ℃ and a curtain gas of 25 psi. Qualitative analysis was finished according to the standards, and the chromatographic peaks were integrated to accomplish quantitative analysis based on a standard curve.

## Supplementary Information

Supplementary Material 1. Figure S1. Comparison of amino acid sequences of DcLcyB1 protein in different carrot varieties. Figure S2. Comparison of amino acid sequences of DcLcyB2 protein in different carrot varieties. Figure S3. Contents of total carotenoid of the carotenoid extraction from *E. coli*. Figure S4. The total carotenoid contents in roots of WT-BHJS, *DcLcyB1*-OE, and *DcLcyB2*-OE lines. Figure S5. Prediction of DcLcyB1 protein interacting proteins. Figure S6. Prediction of DcLcyB2 protein interacting proteins. Figure S7. Contents of total carotenoids in roots of WT-KRD, *dclcyb1*, and *dclcyb2* mutants. Figure S8. Contents of 5 main types of xanthophylls in roots of WT-KRD, *dclcyb1* and *dclcyb2* carrot mutants.

Supplementary Material 2. Table S1. Primers used in this study. Table S2. Qualitative and quantitative analysis of xanthophylls in *DcLcyB1/2*-OE lines. Table S3. Information of target sites of DcLcyB1 and DcLcyB2. Table S4. The changes in amino acid residues in the *dclcyb1* and *dclcyb2* mutants.

## Data Availability

All data generated during this study are included in the published manuscript. All other data are available from the corresponding author.

## Abbreviations

BHJS: Benhongjinshi
BY: Baiyu
CCS: Capsanthin-capsorubin synthase
DAS: Days after sowing
DPP: Deep purple
DW: Dry weight
KRD: Kurodagosun
LC–MS/MS: Liquid chromatograph-tandem mass spectrometer
LcyB: Lycopene β-cyclase
LcyE: Lycopene ɛ-cyclase
LKD: Leikende
MGH: Meiguihong
NJYS: Nanjingyesheng
OE: Overexpression
ORF: Open reading frame
QTH: Qitouhuang
UPLC: Ultra-high performance liquid chromatography
WT: Wild type
