# Supplementary material for: DcLcyB1 and DcLcyB2, two lycopene β-cyclases with partial functional overlap modulate carotene profiles in carrot roots via distinct catalytic properties
Source: Mol Hortic. 2026 Jul 1;6:47. doi: 10.1186/s43897-026-00232-z (PMC13321510; doi:10.1186/s43897-026-00232-z)
Supplement: Supplementary file 1 — Supplementary Material 1. Figure S1. Comparison of amino acid sequences of DcLcyB1 protein in different carrot varieties. Figure S2. Comparison of amino acid sequences of DcLcyB2 protein in different carrot varieties. Figure S3. Contents of total carotenoid of the carotenoid extraction from E. coli. Figure S4. The total carotenoid contents in roots of WT-BHJS, DcLcyB1-OE, and DcLcyB2-OE lines. Figure S5. Prediction of DcLcyB1 protein interacting proteins. Figure S6. Prediction of DcLcyB2 protein interacting proteins. Figure S7. Contents of total carotenoids in roots of WT-KRD, dclcyb1, and dclcyb2 mutants. Figure S8. Contents of 5 main types of xanthophylls in roots of WT-KRD, dclcyb1 and dclcyb2 carrot mutants. [file 43897_2026_232_MOESM1_ESM.pdf]

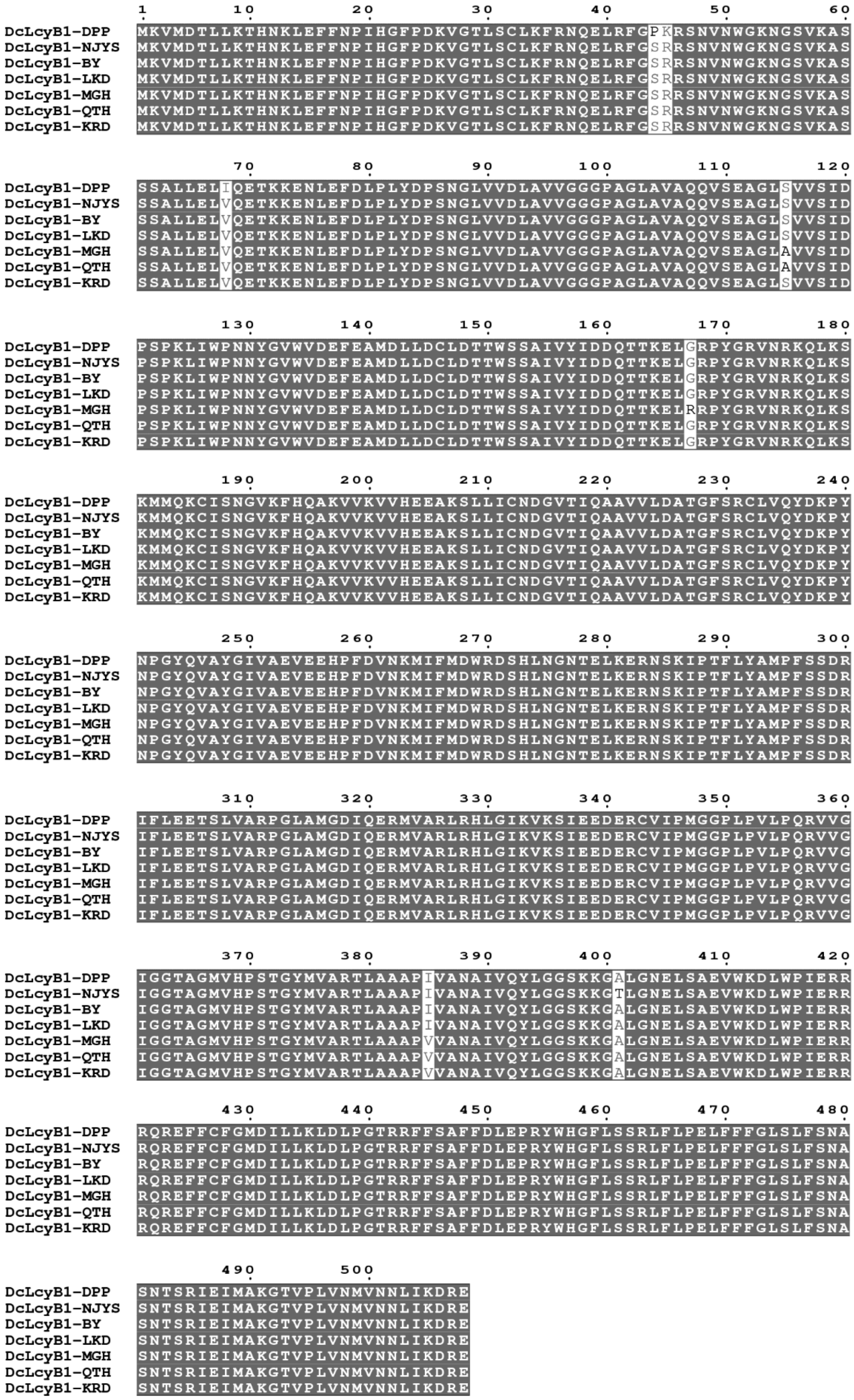

Figure S1. Comparison of amino acid sequences of DcLcyB1 protein in different carrot varieties.

DPP, Deep Purple. NJYS, Nanjingyesheng. BY, Baiyu. LKD, Leikende. MGH, Meiguihong. QTH, Qitouhuang. KRD, Kurodagosun.

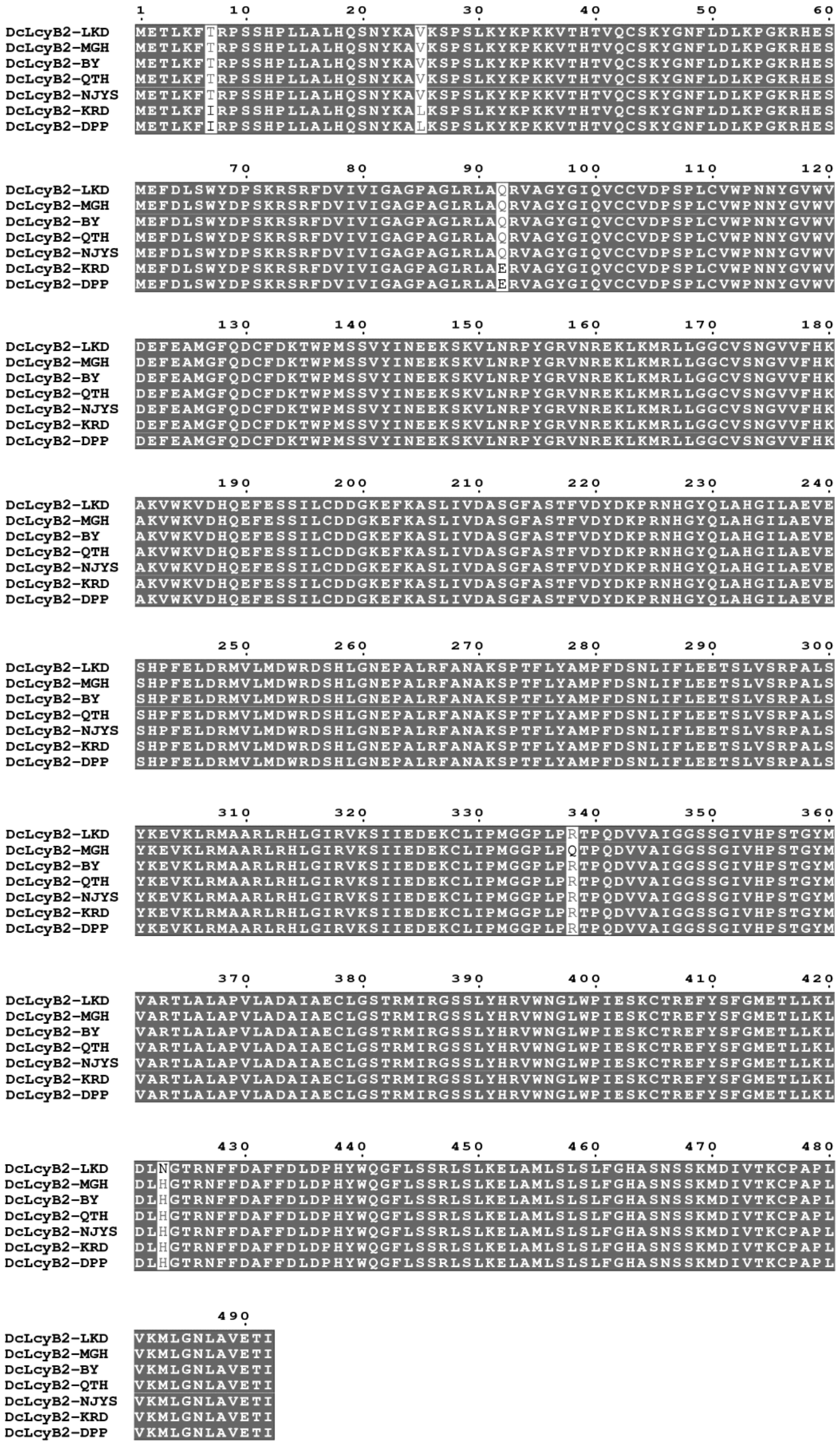

Figure S2. Comparison of amino acid sequences of DcLcyB2 protein in different carrot varieties.

DPP, Deep Purple. NJYS, Nanjingyesheng. BY, Baiyu. LKD, Leikende. MGH, Meiguihong. QTH, Qitouhuang. KRD, Kurodagosun.

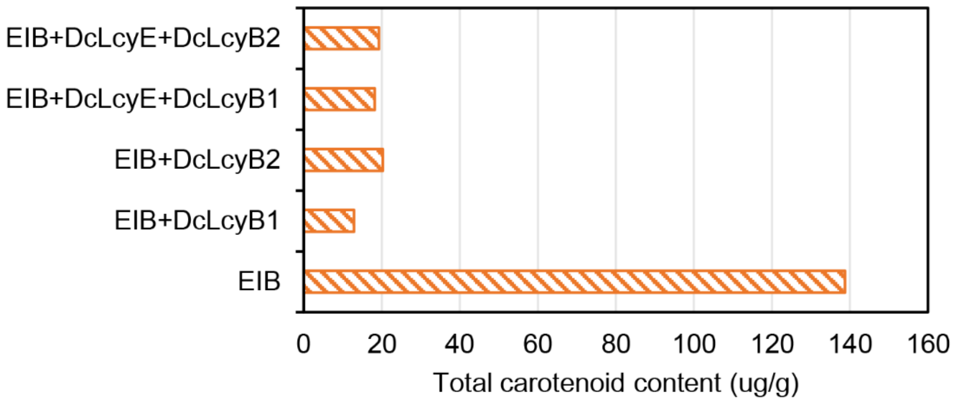

Figure S3. Contents of total carotenoid of the carotenoid extraction from *E. coli*. pACCRT-EIB, EIB.

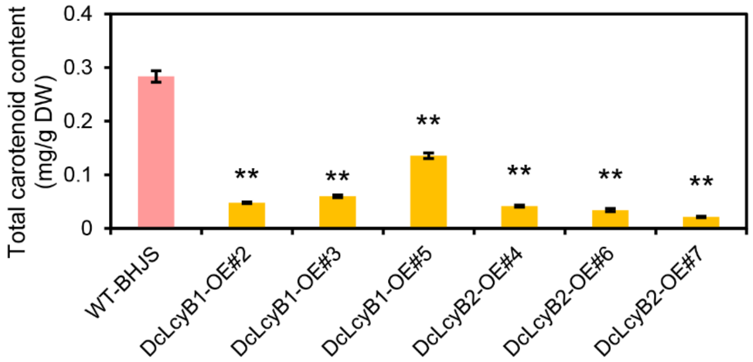

Figure S4. The total carotenoid contents in roots of WT-BHJS, *DcLcyB1*-OE, and *DcLcyB2*-OE lines. '**' represent significant differences in $p < 0.01$ levels between transgenic lines and WT. Dry weight, DW.

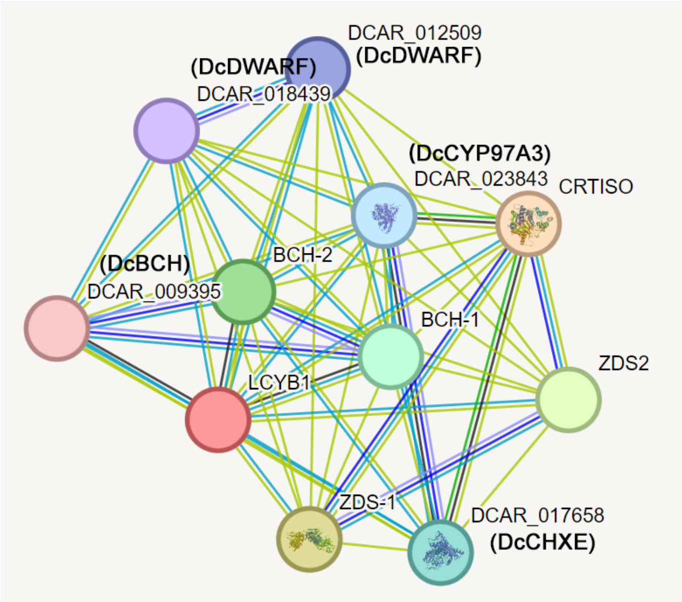

Figure S5. Prediction of DcLcyB1 protein interacting proteins.

BCH, β-carotene hydroxylase; CHXE, carotene ε-monooxygenase; CRTISO, carotenoid isomerase; DWARF, β-carotene isomerase; ZDS, ζ-carotene desaturase.

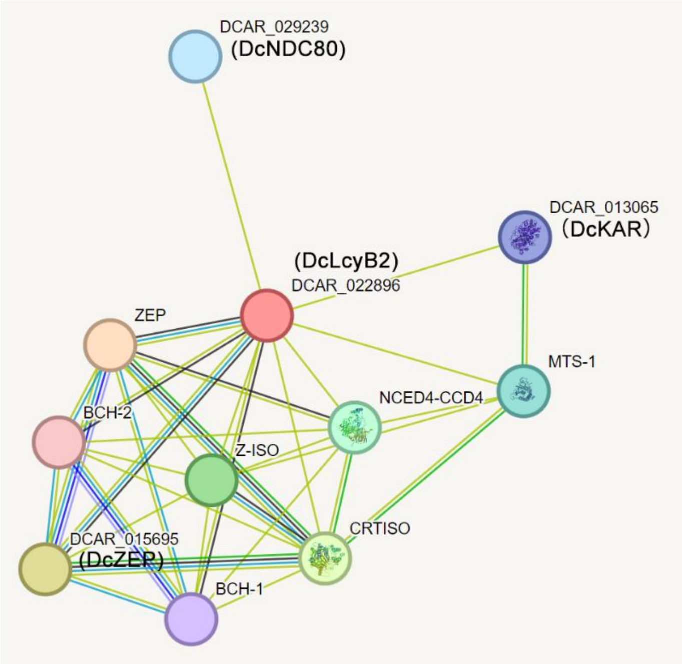

Figure S6. Prediction of DcLcyB2 protein interacting proteins.

BCH, β-carotene hydroxylase; CRTISO, carotenoid isomerase; NCDE-CCD, 9-cis-epoxycarotenoid dioxygenase; MTS, 2-C-methyl-D-erythritol 2,4-cyclodiphosphate synthase; NDC80, kinetochore protein Ndc80; KAR, ketol-acid reductoisomerase (NADP(+)); ZEP, zeaxanthin epoxidase; Z-ISO, ζ-carotene isomerase.

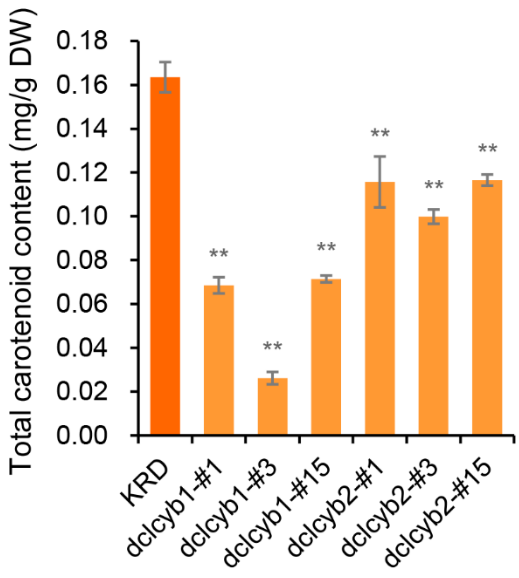

Figure S7. Contents of total carotenoids in roots of WT-KRD, *dclcyb1*, and *dclcyb2* mutants. '**' represent significant differences in $p < 0.01$ levels between transgenic lines and WT.

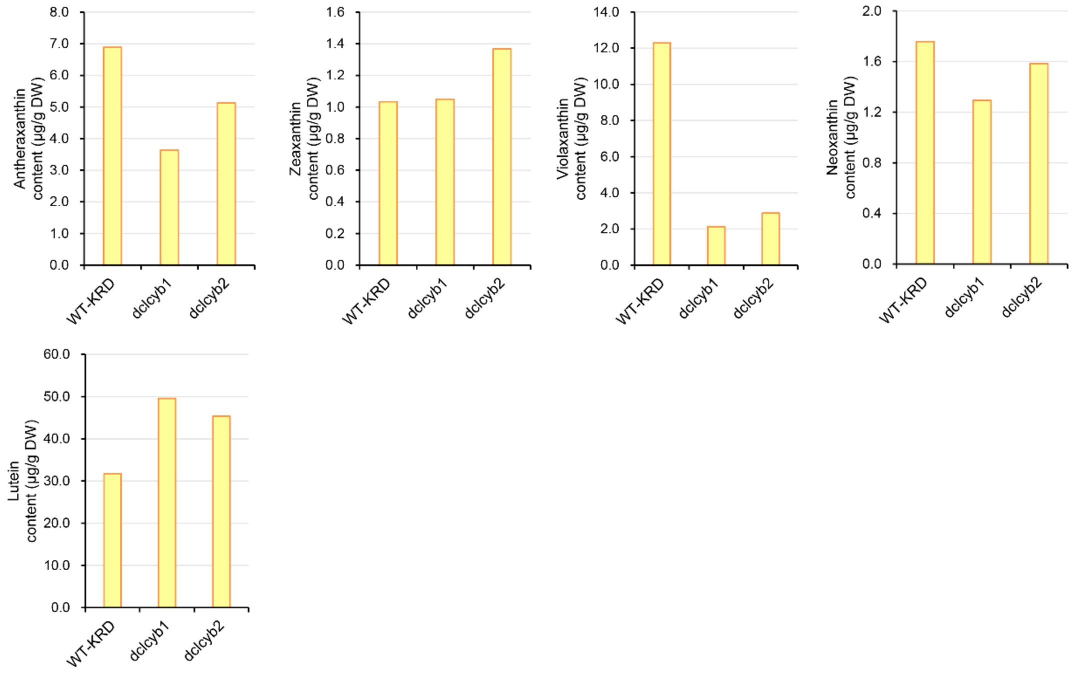

Figure S8. Contents of 5 main types of xanthophylls in roots of WT-KRD, *dclcyb1* and *dclcyb2* carrot mutants.

The WT-KRD sample was taken from 3 different wild-type individual plants. The *dclcyb1* and *dclcyb2* samples were measured by mixing the three test mutant plants *dclcyb1-#1*, *dclcyb1-#3*, *dclcyb1-#15* and *dclcyb2-#1*, *dclcyb2-#3*, *dclcyb2-#5*, respectively.
