# Supplementary material for: DcLcyB1 and DcLcyB2, two lycopene β-cyclases with partial functional overlap modulate carotene profiles in carrot roots via distinct catalytic properties
Source: Mol Hortic. 2026 Jul 1;6:47. doi: 10.1186/s43897-026-00232-z (PMC13321510; doi:10.1186/s43897-026-00232-z)
Supplement: Supplementary file 2 — Supplementary Material 2. Table S1. Primers used in this study. Table S2. Qualitative and quantitative analysis of xanthophylls in DcLcyB1/2-OE lines. Table S3. Information of target sites of DcLcyB1 and DcLcyB2. Table S4. The changes in amino acid residues in the dclcyb1 and dclcyb2 mutants. [file 43897_2026_232_MOESM2_ESM.pdf]

Table S1. Primers used in this study.

Primer name	Primer sequence	Purpose
DcLcyB1-full-F	ATGAAAGTGATGGATACTCTACT	Gene cloning
DcLcyB1-full-R	CTATTCTCTATCTTTGATCAGAT	
DcLcyB2-full-F	ATGGAGACCCTTAAATTTATCAG	
DcLcyB2-full-R	TCAAATAGTCTCAACAGCTAAAT	
DcLcyB1-pGEX-F	GGTCGTGGGATCCCCGAATTCATGAAAGTGATGGATACTCTACT	Prokaryotic expression in *E. coli*
DcLcyB1-pGEX-R	GTCACGATGCGGCCGCTCGAGCTATTCTCTATCTTTGATCAGAT	
DcLcyB2-pGEX-F	GGTCGTGGGATCCCCGAATTCATGGAGACCCTTAAATTTATCAG	
DcLcyB2-pGEX-R	GTCACGATGCGGCCGCTCGAGTCAAATAGTCTCAACAGCTAAAT	
DcLcyB2-1301-F	TTTACAATTACCATGGGATCCATGAAAGTGATGGATACTCTACT	Carrot genetic transformation
DcLcyB2-1301-R	ACCGATGATACGAACGAGCTCCTATTCTCTATCTTTGATCAGAT	
DcLcyB2-1301-F	TTTACAATTACCATGGGATCCATGGAGACCCTTAAATTTATCAG	
DcLcyB2-1301-R	ACCGATGATACGAACGAGCTCTCAAATAGTCTCAACAGCTAAAT	
1301-F	TCATTTGGAGAGGACACGTATTT	Identification of overexpression lines
1301-R	AAGCACAACAAATGGTACAAG	
DcLcyB1-qrt-F	TTGACCTTCCTTTGTATGACCCG	RT-qPCR
DcLcyB1-qrt-R	CTGCCTCAGAAACTTGTTGTGCT	
DcLcyB2-qrt-F	GCGAGGCTCAGACATTTAGGAA	
DcLcyB2-qrt-R	TGCCACCATGTACCCTGTTGAA	
DcLcyE-qrt-F	GTGGCAGGGATTTCTTGGTTCT	
DcLcyE-qrt-R	TTACAAGGGACATTCTCAAGTGGTG	
DcPSY1-qrt-F	GAATGAGGCCTACGATCGGTGT	
DcPSY1-qrt-R	GAATGAGGCCTACGATCGGTGT	
DcPSY2-qrt-F	TGTCAGTTGCTATGTCCTGGATTGT	
DcPSY2-qrt-R	CTTACCCTTCTCAAGTCTGCCTCC	

DcPDS-qrt-F	AAGTCAAGTTTGCGTTGGGTCTC
DcPDS-qrt-R	CGAAAACCTCAGTTGTAACCCGA
DcZDS1-qrt-F	GAGGATGATGATGCTTGTTCGC
DcZDS1-qrt-R	CCGCAAGCCCAGCTCCTATAA
DcZDS2-qrt-F	TATCTATTTTCCGGCCACTTCC
DcZDS2-qrt-R	GCTTTGGTCCACGATAATGTTCTG
DcZISO-qrt-F	GTTTGGAATGGTGACAGAAGGCT
DcZISO-qrt-R	CCTCGAGAATAGCTGCAAATGGTA
DcCRTISO-qrt-F	GCATCTTTGAAACCCCATTACCA
DcCRTISO-qrt-R	GCTTCCTCTTTCTCCTACTCACCC
DcCYP97A3-qrt-F	CATTTCAATCTGGAACTTACATCGC
DcCYP97A3-qrt-R	GGTCTCATTTGGGTTGGGTCC
DcCHXE-qrt-F	AAGCCCAAGCAGAAGTTGATAGAGT
DcCHXE-qrt-R	CTCATTTATGCAGCGTGTCAGGTA
DcCHXB1-qrt-F	GCGAGGAAGAAGTCGGAGAGGT
DcCHXB1-qrt-R	AAGAGAATCTGTAATAAACCGCCAA
DcCHXB2-qrt-F	AGGTTCCTCACTCAGAGATGCTTG
DcCHXB2-qrt-R	ATCTTGCCCAGTACTCCATGCC
DcZEP1-qrt-F	TGGAGCGTGCTACAAAAGGAGA
DcZEP1-qrt-R	TGGAGCGTGCTACAAAAGGAGA
DcZEP2-qrt-F	GTGGTTCAGCAGGCTTGGA
DcZEP2-qrt-R	GCGAGGTAGATTCTGTTATGAGTAA
DcVDE-qrt-F	TCCATTACGAAGATGACTGT
DcVDE-qrt-R	TTCTCCACCTCCTGTTCTA
DcNXS1-qrt-F	AATATCTATTGCTGGTCCGACTATG
DcNXS1-qrt-R	TGAATACACTTCTTGGTCAGAGTAG

DcNXS2-qrt-F	TGAATACACTTCTTGGTCAGAGTAG
DcNXS2-qrt-R	TCCATTCGGTTCTACTCTTC
DcCCD1a-qrt-F	GGGTGACAGAGCACGAGAAA
DcCCD1a-qrt-R	GCACAGAAGCGTCATACATC
DcCCD4-qrt-F	AGACCACCAACCACTACTCCTACC
DcCCD4-qrt-R	GCCGTGGATGACCTCGCATT
DcCCD7-qrt-F	ACATGATTCTGCGACGGATGAGTG
DcCCD7-qrt-R	CCTCTTCGGCGGCATATTGAACA
DcCCD8-qrt-F	AGGCAGCATTGAATCCAGATGAACA
DcCCD8-qrt-R	ACAGCACCGCAAGCATAAGCATA
DcActin-qrt-F	CGGTATTGTGTTGGACTCTGGTGAT
DcActin-qrt-R	CAGCAAGGTCAAGACGGAGTATGG

Table S2. Qualitative and quantitative analysis of xanthophylls in DcLcyB1/2-OE lines

Compounds	Class	Q1 (Da)	Q3 (Da)	Molecular Weight	Ion mode	Ionization model	Formula	Content		
								WT-BHJS	DcLcy B1-OE	DcLcy B2-OE
β-cryptoxanthin oleate	xanthophylls	817.8	535.4	816.8	positive	[M+H]+	$C_{58}H_{88}O_2$	N/A	N/A	0.03641
zeaxanthin-palmitate-stearate	xanthophylls	1074.1	789.8	1073.1	positive	[M+H]+	$C_{74}H_{120}O_4$	N/A	N/A	0.0063
lutein oleate	xanthophylls	815.7	533.4	832.7	positive	[M+H-18]+	$C_{58}H_{88}O_3$	N/A	N/A	0.0734
zeaxanthin palmitate	xanthophylls	807.8	551.5	806.8	positive	[M+H]+	$C_{56}H_{86}O_3$	N/A	N/A	0.0634
5,6epoxy-lutein-caprate-palmitate	xanthophylls	805.4	549.4	976.4	positive	[M+H-172]+	$C_{66}H_{104}O_5$	0.0450	N/A	N/A
violaxanthin-myristate-caprate	xanthophylls	965.7	947.8	964.7	positive	[M+H]+	$C_{64}H_{100}O_6$	N/A	0.0766	N/A
violaxanthin dilaurate	xanthophylls	966.7	948.8	965.7	positive	[M+H]+	$C_{64}H_{101}O_6$	0.0258	N/A	N/A
rubixanthin palmitate	xanthophylls	791.7	535.4	790.7	positive	[M+H]+	$C_{56}H_{86}O_2$	0.0237	0.0366	0.4610
zeaxanthin dipalmitate	xanthophylls	789.5	533.5	1045.1	positive	[M+H-256]+	$C_{72}H_{116}O_4$	N/A	N/A	0.2069
rubixanthin myristate	xanthophylls	763.5	533.5	762.5	positive	[M+H]+	$C_{54}H_{82}O_2$	N/A	0.0085	N/A
neochrome palmitate	xanthophylls	821.7	565.5	838.7	positive	[M+H-18]+	$C_{56}H_{86}O_5$	0.0414	0.0336	N/A

β-cryptoxanthin laurate	xanthophylls	735.8	535.5	734.8	positive	[M+H]+	C52H78O2	0.0324	0.0583	0.0863
β-cryptoxanthin palmitate	xanthophylls	791.9	535.5	790.9	positive	[M+H]+	C56H86O2	0.0228	0.0219	0.4318
lutein palmitate	xanthophylls	789.8	533.5	806.8	positive	[M+H-18]+	C56H86O3	0.0836	0.0874	1.1212
lutein dilaurate	xanthophylls	733.5	533.3	933.5	positive	[M+H-201]+	C64H101O4	0.1190	0.1832	0.1196
lutein dimyristate	xanthophylls	761.8	533.5	988.8	positive	[M+H-228]+	C68H108O4	0.1431	0.1380	0.9069
lutein dipalmitate	xanthophylls	789.8	533.5	1044.8	positive	[M+H-256]+	C72H116O4	0.0728	0.0892	0.7190
violaxanthin myristate	xanthophylls	811.8	793.7	810.8	positive	[M+H]+	C54H82O5	1.037	0.5526	1.2633
violaxanthin-myristate-palmitate	xanthophylls	1050	793.8	1049.0	positive	[M+H]+	C70H112O6	0.0540	0.0359	0.1430
antheraxanthin	xanthophylls	585.5	175.4	584.4	positive	[M+H]+	C40H56O3	4.6380	7.8759	9.7532
zeaxanthin	xanthophylls	569.4	477.5	568.4	positive	[M+H]+	C40H56O2	2.2260	9.3860	6.3655
violaxanthin	xanthophylls	601.4	221	600.4	positive	[M+H]+	C40H56O4	1.3452	0.7945	3.3158
neoxanthin	xanthophylls	601.4	565.5	600.4	positive	[M+H]+	C40H56O4	0.6427	0.3735	0.9595
lutein	xanthophylls	551.5	175.4	568.4	positive	[M+H-18]+	C40H56O2	65.6173	110.6773	100.4515
β-cryptoxanthin	xanthophylls	553.5	177.4	552.4	positive	[M+H]+	C40H56O	8.1617	0.8083	1.2900
8'-apo-beta-carotenal	xanthophylls	417.3	325.3	416.6	positive	[M+H]+	C30H40O	0.1536	0.0171	0.0338
α-cryptoxanthin	xanthophylls	553.5	123.1	552.4	positive	[M+H]+	C40H56O	0.5367	0.1739	0.1212
echinenone	xanthophylls	551.6	203.1	550.9	positive	[M+H]+	C40H54O	0.1973	0.0135	0.0278
lutein caprate	xanthophylls	705.7	533.5	722.7	positive	[M+H-18]+	C50H74O3	0.0274	0.0120	0.0457
lutein distearate	xanthophylls	817.9	533.5	1100.9	positive	[M+H-284]+	C76H124O4	0.0380	0.0251	0.2825

rubixanthin laurate	xanthophylls	735.6	535.4	734.6	positive	[M+H]+	C52H78O2	0.0453	0.0666	0.0822
violaxanthin dibutyrate	xanthophylls	741.6	653.5	740.6	positive	[M+H]+	C48H68O6	0.0070	0.0696	0.058
violaxanthin laurate	xanthophylls	783.7	583.4	800.7	positive	[M+H-18]+	C52H80O6	0.0560	0.0412	0.0623
violaxanthin-myristate-laurate	xanthophylls	993.8	975.7	992.8	positive	[M+H]+	C66H104O6	0.2526	0.2191	0.3081
violaxanthin dimyristate	xanthophylls	1021.8	793.7	1020.8	positive	[M+H]+	C68H108O6	0.0138	0.0213	0.0116
β-cryptoxanthin myristate	xanthophylls	763.9	535.5	762.9	positive	[M+H]+	C54H82O2	0.0502	0.1016	0.1624
antheraxanthin dipalmitate	xanthophylls	1061.0	805.0	1060.0	positive	[M+H]+	C72H115O5	0.0161	N/A	0.1229
lutein dioleate	xanthophylls	815.7	533.4	1096.7	positive	[M+H-282]+	C76H120O4	0.0074	N/A	0.0339
violaxanthin dipalmitate	xanthophylls	1077.9	821.7	1076.9	positive	[M+H]+	C72H116O6	0.0212	N/A	0.1309
lutein stearate	xanthophylls	817.8	533.5	834.8	positive	[M+H-18]+	C58H90O3	0.0333	N/A	0.0711
violaxanthin palmitate	xanthophylls	839.8	821.8	838.8	positive	[M+H]+	C56H86O5	N/A	N/A	1.3802
canthaxanthin	xanthophylls	565.5	203.3	564.8	positive	[M+H]+	C40H52O2	0.0022	0.0004	0.0006

Note: ‘N/A’ represents not detected.

Table S3. Information of target sites of *DcLcyB1* and *DcLcyB2*.

Gene	Number	Sequence	Position	Direction	GC content (%)	Promoter
DcLcyB1	T1	CGAAACCAGGAGCTGAGGTT <u>TGG</u>	106 - 125	+	55.0	*AtU3d*
	T2	TGGAGGTCCTGCAGGGTTAG <u>CGG</u>	291 - 310	+	60.0	*AtU6-1*
	T3	ACAGGCAGAGGCCCTCCCAT <u>TGG</u>	1058 - 1039	-	65.0	*AtU3b*
	T4	CTGGAACCTCGCTATTGGCA <u>TGG</u>	1351 - 1370	+	55.0	*AtU6-29*
DcLcyB2	T1	GTGATCGGAGCTGGTCCAGC <u>CGG</u>	238 - 257	+	65.0	*AtU6-1*
	T2	TAAGGCCAAGGTTTGGAAGG <u>TGG</u>	537 - 556	+	50.0	*AtU3b*
	T3	CGAGGCAAGGGTCCTCCCAT <u>TGG</u>	1013 - 994	-	65.0	*AtU6-29*
	T4	CTATTGAGAGCAAGTGCACC <u>AGG</u>	1199 - 1218	+	50.0	*AtU6-1*

Note: Underlines represent PAM sequences.

Table S4. The changes in amino acid residues in the *dclcyb1* and *dclcyb2* mutants.

Mutants	Target	Position	Mutation type	Nucleotide sequence	Amino acid residues
dclcyb1-#1	T3	1053	Substitution	CTG → CTA	Leu → Leu (Downstream frameshift)
dclcyb1-#3	T3	1053	Substitution	CTG → CTA	Leu → Leu (Downstream frameshift)
dclcyb1-#3	T4	1353	Substitution	CTG → CTA	Leu → Leu (Downstream frameshift)
dclcyb1-#15	T1	122	Insertion	AGG → AGAG	Arg → Arg → Premature termination
dclcyb1-#15	T2	292	Substitution	TGG → GGG	Trp → Gly
dclcyb1-#15	T2	304	Substitution	AGG → GGG	Arg → Gly
dclcyb2-#1	T1	250	Substitution	TGG → TAG	Trp → Premature termination
dclcyb2-#1	T4	1210	Deletion	AAG → -AG	Lys → Ser → Premature termination
dclcyb2-#3	T3	999	Deletion	AGGACC → ------	Gly Pro → -- (Frameshift)
dclcyb2-#5	T1	245	Substitution	GGA → GAA	Gly → Glu
